# Gene Isoform Specificity through Enhancer-Associated Antisense Transcription

**DOI:** 10.1371/journal.pone.0043511

**Published:** 2012-08-24

**Authors:** Courtney S. Onodera, Jason G. Underwood, Sol Katzman, Frank Jacobs, David Greenberg, Sofie R. Salama, David Haussler

**Affiliations:** 1 Department of Biomolecular Engineering, University of California Santa Cruz, Santa Cruz, California, United States of America; 2 Center for Biomolecular Sciences and Engineering, University of California Santa Cruz, Santa Cruz, California, United States of America; 3 Howard Hughes Medical Institute, University of California Santa Cruz, Santa Cruz, California, United States of America; 4 Department of Molecular, Cell and Developmental Biology, University of California Santa Cruz, Santa Cruz, California, United States of America; University of Crete, Greece

## Abstract

Enhancers and antisense RNAs play key roles in transcriptional regulation through differing mechanisms. Recent studies have demonstrated that enhancers are often associated with non-coding RNAs (ncRNAs), yet the functional role of these enhancer:ncRNA associations is unclear. Using RNA-Sequencing to interrogate the transcriptomes of undifferentiated mouse embryonic stem cells (mESCs) and their derived neural precursor cells (NPs), we identified two novel enhancer-associated antisense transcripts that appear to control isoform-specific expression of their overlapping protein-coding genes. In each case, an enhancer internal to a protein-coding gene drives an antisense RNA in mESCs but not in NPs. Expression of the antisense RNA is correlated with expression of a shorter isoform of the associated sense gene that is not present when the antisense RNA is not expressed. We demonstrate that expression of the antisense transcripts as well as expression of the short sense isoforms correlates with enhancer activity at these two loci. Further, overexpression and knockdown experiments suggest the antisense transcripts regulate expression of their associated sense genes via *cis*-acting mechanisms. Interestingly, the protein-coding genes involved in these two examples, Zmynd8 and Brd1, share many functional domains, yet their antisense ncRNAs show no homology to each other and are not present in non-murine mammalian lineages, such as the primate lineage. The lack of homology in the antisense ncRNAs indicates they have evolved independently of each other and suggests that this mode of lineage-specific transcriptional regulation may be more widespread in other cell types and organisms. Our findings present a new view of enhancer action wherein enhancers may direct isoform-specific expression of genes through ncRNA intermediates.

## Introduction

Many studies have revealed that antisense transcription (transcription from the opposite strand of a protein-coding or sense gene) is widespread throughout the genome. This finding was first made possible through large-scale cDNA and EST sequencing efforts and has sparked interest in uncovering functional roles for these antisense transcripts, often termed Natural Antisense Transcripts, or NATs (reviewed in [1]). In mouse [2] and in human [3], thousands of sense/antisense (S/AS) pairs of transcripts have been revealed in this manner. Additionally, through subsequent microarray analysis, many S/AS pairs have been found to show correlated expression, with concordant expression (positive expression correlation) more common than reciprocal expression (negative expression correlation) [2]. This correlated expression suggests that the antisense transcripts in S/AS pairs may often be involved in regulation of their sense partners.

Indeed, antisense transcripts have been demonstrated to exhibit regulatory activity in several instances. Transcriptional interference represents one known mechanism. In this phenomenon, the act of transcription from one strand prevents the initiation or elongation of transcription from the opposite strand through steric hindrance (reviewed in [4]). Quite recently, Morrissy et al. used microarrays to show that expression of antisense transcripts strongly correlates with alternative splicing of sense targets [5]. The authors propose that antisense transcripts may regulate alternative splicing of their sense targets through base-pairing of the complementary regions, resulting in splice site masking or the production of endogenous siRNAs, or through transcriptional interference.

Of course, gene regulation also occurs at the DNA level, particularly through enhancers. Enhancers have traditionally been understood to be *cis*-acting DNA elements which act as binding sites for transcription factors or activator proteins that in turn recruit or stabilize RNA polymerase II transcription at specific promoters (reviewed in [6], [7]). Interestingly, recent work has demonstrated widespread transcription at enhancers [8], [9]. While it has long been known that enhancers can occur in introns of genes, the finding that extragenic enhancers are often transcribed raises questions regarding the functions of these enhancer-associated transcripts. Recently, short, non-polyadenylated RNA pol II transcripts arising bidirectionally from active enhancers have been reported [9], but functional roles for these transcripts, termed “eRNAs,” have not been identified. Indeed, as bidirectional transcription has also been demonstrated at promoters [10]–[12], it is not unreasonable to postulate these eRNAs could largely be transcriptional noise. Among the few enhancer-associated RNAs to be characterized at a functional level, the polyadenylated ncRNA Evf-2 is transcribed from an enhancer and interacts with this same enhancer to drive transcription of homeodomain proteins DLX5 and DLX6 [13]. Evf-2 currently represents only an isolated example of an enhancer-associated RNA, yet this finding suggests that enhancers and ncRNAs may cooperate on a larger scale to achieve finely-tuned gene regulation.

In this study we functionally characterize two specific novel enhancer-associated transcripts discovered through RNA-Sequencing (RNA-Seq) of mouse embryonic stem cells (mESCs) and their derived neural precursors (NPs). Each of these RNAs originates within and is expressed antisense to a known protein-coding gene. Further, the expression of each antisense RNA is correlated with an active enhancer, located by P300 binding. Finally, in each case the antisense transcript is expressed concomitantly with the sense transcript, but a shorter isoform of the sense gene appears to be preferentially expressed when the antisense transcript is also expressed. From these findings we propose a model in which gene isoform specificity may be achieved through enhancer-associated antisense RNAs. This model challenges the long-held view that enhancers act strictly on promoters of protein-coding genes to accomplish gene regulation.

## Results

### RNA-Sequencing of two timepoints in mESC neural differentiation reveals novel enhancer-associated RNAs

**Figure 1:**
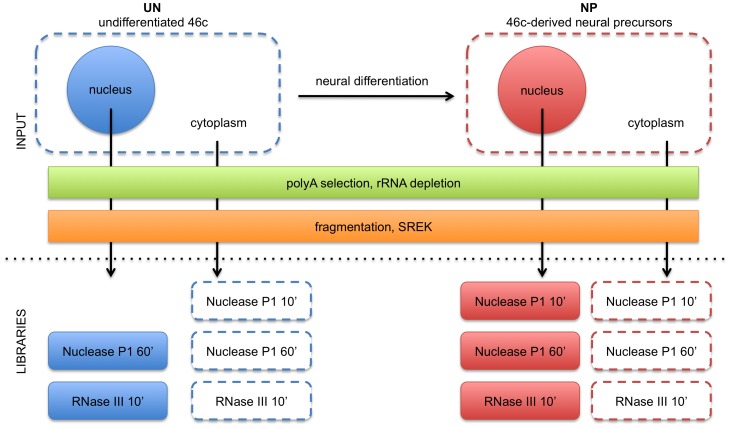
RNA-Seq libraries generated. Polyadenylated nuclear and cytoplasmic RNAs from undifferentiated (UN) mESCs and mESC-derived day 5 NPs (NP) were fragmented with either Nuclease P1 or RNase III before preparation of libraries with the Applied Biosystems (now Life Technologies) Small RNA Expression Kit (SREK) for sequencing with the SOLiD V3 platform. For most subsequent analyses, reads from libraries corresponding to the same cell and compartment type were amassed together and treated as a single library (e.g., reads from UN nuclear Nuclease P1 60′ and UN nuclear RNase III 10′ were treated together as the UN nuclear library.) See Methods and Text 8 for more details. Hereafter we will use the notations UnNuc, UnCyt, NPNuc, and NPCyt to refer to undifferentiated nuclear, undifferentiated cytoplasmic, day 5 NP nuclear, and day 5 NP cytoplasmic RNAs/libraries/reads, respectively.

To uncover novel enhancer-associated RNAs, we sequenced the polyA+ transcriptomes of undifferentiated mESCs (line 46C) and their derived NPs (Text S1). We fractionated the cells in each population to their nuclear and cytoplasmic components [14] prior to RNA extraction, polyA selection, rRNA removal, and enzymatic fragmentation for sequencing with the SOLiD (Life Technologies) V3 platform (Text S1), yielding 50 bp single-end reads. In total, we sequenced 11 barcoded libraries (Fig. 1), from which we obtained 190 million reads (Table S1). We mapped these reads against the mm9 assembly of the mouse genome on the UCSC Genome Browser [15] using the SOLiD Corona pipeline (Fig. S1; Text S1) and obtained an overall mapping rate of 36.5% (Table S1). For the remainder of our analyses, we grouped reads from the same cell/compartment type together to simplify our libraries to 4 main types, UnNuc, UnCyt, NPNuc, and NPCyt, corresponding to undifferentiated nuclear, undifferentiated cytoplasmic, NP nuclear, and NP cytoplasmic, respectively.

Our final libraries and read mappings appear to be high-quality by several measures. First, known nuclear and cytoplasmic RNAs show enrichment as expected in our libraries (Fig. S2; Text S1). Next, we detect even lowly-expressed housekeeping genes [16] in all libraries, indicating we have sequenced at sufficient depth to uncover even lowly-expressed novel transcripts (Fig. S3; Text S1). Finally, our libraries compare well with a previous microarray assessment of mESC neural differentiation [17], indicating our libraries are good representations of the transcriptional activity in each cell population (Figs. S4, S5; Text S1).

To find novel transcripts associated with enhancers, we first assembled transcripts from our RNA-Seq read mappings through a method based on the Cufflinks program [18] (Text S1; see also Figs. S6, S7, S8, S9, S10, and Table S2). We then compiled information from several genome-wide enhancer studies. We included chromatin immunoprecipitation sequencing (ChIP-Seq) data for P300, a histone acetyltransferase and known enhancer-associated protein, from mESCs [19], human ESCs [20], and mouse brain and limb structures [21]. We additionally included ChIP data for a known enhancer-associated histone modification, histone 3 lysine 4 monomethylation (H3K4Me1), from mESCs and mESC-derived neural precursors [22], [23]. Finally, we included data for enhancers verified through a transient-transgenic mouse embryo assay [24], [25]. These sets reflected the currently available genome-wide experimentally-supported enhancers in analogous mouse and human cell types. From our RNA-Seq data we discovered a total of 18 putative novel enhancer-associated RNAs (Table S3) meeting the following criteria: the RNAs are novel and do not appear to be part of a known transcript, the RNAs are expressed when a putative enhancer is apparently active, and the putative enhancer is located within or just upstream of the mature transcript. The remainder of this paper focuses on two candidates which appear to regulate expression of associated genes via a common mechanism.

### Novel enhancer-associated RNAs that correlate with gene isoform choice

**Figure 2:**
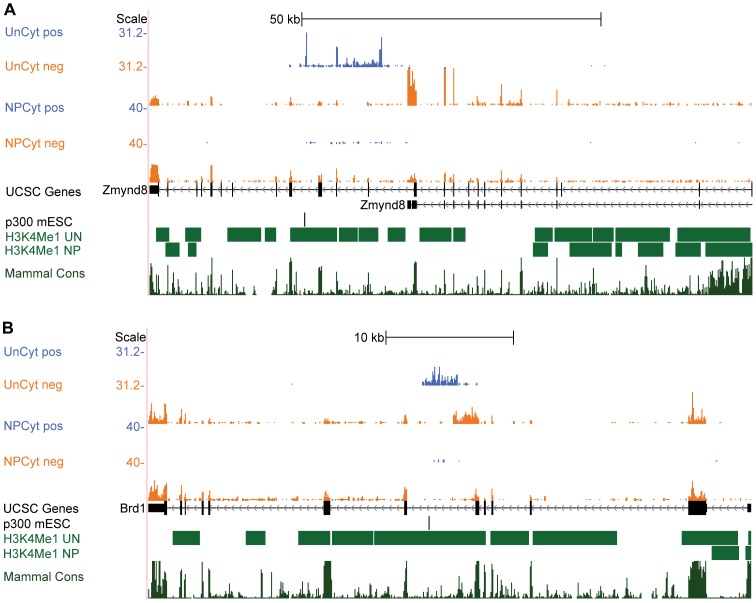
Expression of novel enhancer-associated antisense transcripts appears to correlate with short sense isoforms. UCSC Genome Browser views of novel antisense transcription within known sense genes Zmynd8 (A) and Brd1 (B). At top, blue coverage tracks indicate the number of uniquely placed unspliced reads mapped per base on the positive strand; orange coverage tracks indicate the same for the negative strand. In each case, there is novel positive strand (blue) transcription specific to undifferentiated mESCs and previously known coding genes transcribed on the negative strand (orange). Coverage track heights are scaled according to the number of reads mapped for each RNA-Seq library. Also shown are UCSC Genes [28], along with P300 binding data (ChIP-Seq peaks) from undifferentiated mESCs [19] and H3K4Me1 data (ChIP-Seq peaks) from mESCs and neural precursors [23]. Mammalian conservation (PhastCons) is indicated at bottom [58], [59].

Among our 18 candidate novel enhancer-associated RNAs we found two which appear to correlate with isoform-specific expression of associated genes. In the first case, the novel transcription occurs in undifferentiated mESCs, antisense to known protein-coding gene Zmynd8 (Figs. 2A, S11). We have named this new transcript Zmynd8as because of its antisense nature. Through Rapid Amplification of cDNA Ends (RACE; [26], [27]) and subsequent cloning we characterized Zmynd8as as an 809-nucleotide spliced transcript containing 3 exons (Fig. S11). Interestingly, the second exon of Zmynd8as overlaps an exon of sense Zmynd8. Our RNA-Seq data indicates Zmynd8 is expressed in both undifferentiated mESCs and in NPs, but when Zmynd8as is expressed as well, it appears the major expressed isoform of Zmynd8 ends just before the 3′ end of Zmynd8as (Figs. 2A, S11). Using 3′ RACE, we confirmed the short isoform of Zmynd8 (which we will hereafter refer to as Zmynd8-short) has a structure very similar to the spliced short isoform shown in the UCSC Genes track [28] (Fig. S11). However, examination of the apparent final splice sites in Zmynd8-short as well as examination of the genomic DNA for the 46C cell line reveals this region does not represent a true intron in Zmynd8-short, but a gap in the 46C genomic sequence relative to the reference mouse genome (Text S1). This structure is also observed in EST evidence (Genbank accessions AV512491, from mESCs, and BC023300, from mouse liver). Finally, Chen et al. [19] report a P300 ChIP-Seq peak at the 5′ end of Zmynd8as in undifferentiated mESCs, suggesting this region is active as an enhancer in mESCs. Though NPs were not examined in Chen's study, H3K4Me1 data [22], [23] indicates this putative enhancer may be specific to mESCs, as there is a broad loss of this enhancer-associated histone mark in NPs relative to mESCs (Figs. 2A, S11).

Zmynd8as appears to be noncoding, due to both its general lack of conservation (Figs. 2A, S11) and its lack of a sizeable open reading frame (ORF). With the exception of its second exon, which overlaps a coding exon of Zmynd8, Zmynd8as sequence appears to be murine-specific (Fig. S11). The largest ORF found for Zmynd8as is only 243 nucleotides, covering relatively little of the Zmynd8as transcript, and would produce an 80-amino acid protein containing no known functional domains (determined by InterProScan, [29], [30]). BLAST [31] of this putative protein to the NR database reveals that only the 40 C-terminal residues have weak hits to three uncharacterized mouse proteins. Further, the start codon of this putative ORF is not predicted to possess a true Kozak (translation initiation) sequence, as scored by WeakAUG [32]. Finally, Zmynd8as is scored as noncoding by Kong's Coding Potential Calculator [33]. For these reasons we believe Zmynd8as to be noncoding.

Similar to Zmynd8as, in our second case we again find novel transcription occurring in undifferentiated mESCs, antisense to the known protein-coding gene Brd1 (Figs. 2B, S12). Again, because of its antisense nature, we have named this second novel transcript Brd1as. With RACE and subsequent cloning, we characterized Brd1as as a 1,774-nucleotide unspliced transcript (Fig. S12). Based on our RNA-Seq data, Brd1 is expressed in both undifferentiated mESCs and in NPs, but when Brd1as is expressed, it appears that a shorter isoform of Brd1 is expressed, a finding we confirmed with 3′ RACE (Fig. S12). Again a P300 ChIP-Seq peak is reported at the 5′ end of Brd1as in mESCs [19], and again there is a broad loss of H3K4Me1 at this locus in neural precursors relative to undifferentiated mESCs [22], [23] (Figs. 2B, S12).

As with Zmynd8as, Brd1as appears to be noncoding for several reasons. First, the Brd1as sequence is poorly conserved (Figs. 2B, S12). The largest ORF found in Brd1as is only 108 bp, for a protein of only 35 amino acids that contains no known functional domains per InterProScan [29], [30] and has no hits to known proteins per BLAST [31]. The start codon of this putative ORF is also not predicted to possess a true Kozak sequence per WeakAUG [32]. Finally, Brd1as is scored as noncoding by Kong's Coding Potential Calculator [33].

To summarize, both Zmynd8as and Brd1as are expressed preferentially in undifferentiated mESCs, are expressed antisense to known protein-coding genes, and are associated with P300 binding sites in mESCs. Additionally, in each case expression of the antisense transcript appears to correlate with expression of a shorter isoform of the associated sense protein-coding gene. Note that because our library preparation protocol included a polyA selection step and because our 5′ RACE protocol will amplify only transcripts possessing 5′ 7-methyl-G caps, Zmynd8as and Brd1as are likely fully processed products of pol II transcription. Thus, while Zmynd8as and Brd1as expression is reminiscent of the bidirectional eRNAs originating from enhancers reported by Kim et al. [9], we believe that the these two transcripts represent a class distinct from the short, non-polyadenylated transcripts generalized by Kim.

### Enhancer activity correlates with antisense transcription and short sense isoform

**Figure 3:**
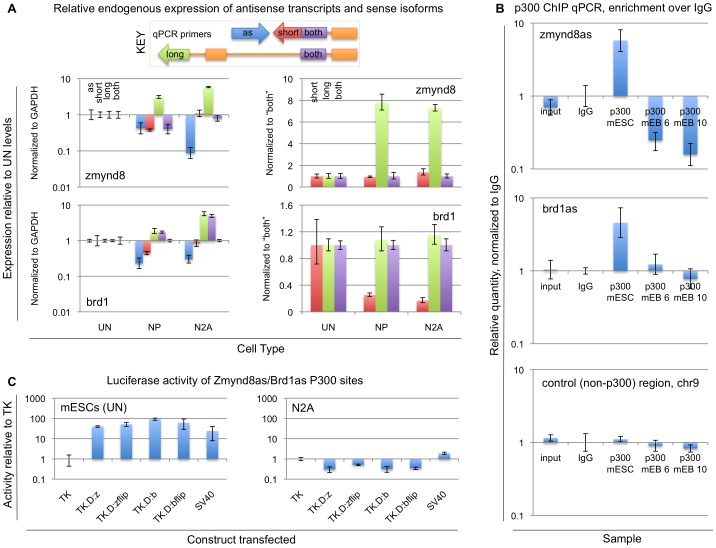
Antisense and short sense isoform expression correlates with enhancer activity. (A) Endogenous expression levels of Zmynd8- and Brd1-associated transcripts in UN, NP, and N2A cells, measured by quantitative RT-PCR, using primers specific to the antisense transcripts (“as”, blue bars), the short sense isoforms (“short”, red bars), the long sense isoforms (“long”, green bars), or both sense isoforms (“both”, purple bars). Expression is shown relative to the levels observed in UN, normalized to GAPDH (left panels), Zmynd8-both (upper right), or Brd1-both (lower right). Chart coloring is also indicated in the key. (B) P300 enrichment at Zmynd8as and Brd1as sites is specific to mESCs. Chromatin immunoprecipitation was performed on mESCs, mEB 6 or mEB 10 NPs with either a P300 or IgG antibody. Relative enrichments of putative P300-bound DNA from Zmynd8as (top), Brd1as (middle), or a putative non-P300 bound site (bottom) over the IgG control (from mESCs) were measured with quantitative PCR. Input (non-antibody-selected, from mESCs) DNA is also shown as an additional control. (C) Test of enhancer activity in undifferentiated mESCs and N2A. P300 sites confirmed in (B) were cloned in both orientations downstream of a luciferase reporter driven by a thymidine kinase (TK) promoter. These constructs were transfected into undifferentiated mESCs (UN; left) or N2A (right), along with the promoter-alone vector, TK, and an alternate promoter-containing vector, SV40. TK.D:z, Zmynd8as P300 site cloned downstream of luciferase with TK promoter; TK.D.zflip, the same site in reverse orientation cloned downstream of luciferase with TK promoter; similarly for TK.D:b and TK.D:bflip for the Brd1as P300 site.

To examine these two antisense ncRNA cases further, we first asked whether the expression of Zmynd8as, Brd1as and their associated short sense isoforms is unique to undifferentiated mESCs in our neural differentiation setting. Using quantitative RT-PCR (qRT-PCR), we measured the endogenous expression of Zmynd8as, Brd1as, and the various isoforms of Zmynd8 and Brd1 in undifferentiated mESCs (UN), NPs (NP), and N2A (Neuro-2A), a mouse neuroblastoma cell line (Fig. 3A). Expression of Zmynd8as and Brd1as is sharply reduced in N2A relative to undifferentiated mESCs, as is also the case in NP, as expected from our RNA-Seq data. Note that in each case the sense isoform abundances in N2A correlate well with those in NP, in contrast to mESCs. Note also that in this assay increased expression of the long isoform of Zmynd8 correlates with decreased expression of Zmynd8as, and similarly, the short isoform of Brd1 shows striking downregulation with decreased expression of Brd1as.

We next examined potential enhancer activity at the 5′ regions of Zmynd8as and Brd1as. We asked whether the P300 binding sites found in the literature were specific to undifferentiated mESCs in the neural differentiation process, as suggested by H3K4Me1 data (Fig. 2). We performed qPCR on P300-selected ChIP DNA from undifferentiated mESCs (line 46C) and two stages of derived neural precursors, mEB 6 and mEB 10 [34]. We found clear enrichment of P300 binding at the Zmynd8as and Brd1as loci as reported by Chen et al. [19] in undifferentiated mESCs (Fig. 3B). Notably, this enrichment is lost upon neural differentiation, consistent with the loss of H3K4Me1 along these regions in Mikkelsen's data [23] shown in Figure 2. Thus we confirm the endogenous Zmynd8as and Brd1as loci appear to be mESC-specific enhancers. Moreover, this enhancer activity does not appear to be limited to the 46C cell line, as Chen's P300 data is from the E14 cell line and Mikkelsen's H3K4Me1 data is from V6.5 mESCs.

To verify that these confirmed P300-enriched regions can indeed upregulate transcription in their predicted cell types in a manner consistent with enhancer activity, we tested these regions in an *in-vitro* luciferase reporter assay (Fig. 3C). We cloned each region in both orientations downstream of a luciferase reporter driven by a thymidine kinase (TK) promoter, and compared the luciferase activity of cells transfected with these constructs against the activity of cells transfected with the TK-only vector. As a positive control, we also tested a construct using an SV40 promoter to drive luciferase. The SV40 promoter constitutively drives expression at a higher rate than the TK promoter in a wide variety of cell types, though the exact magnitude of this difference varies between cell types. From these experiments, we found that in either orientation the Zmynd8as and Brd1as P300 sites show strong upregulation of luciferase in undifferentiated mESCs, consistent with the P300 and H3K4Me1 data. The orientation-independence of these effects, combined with the ability of these regions to upregulate transcription from a position downstream of the reporter, demonstrates these regions exhibit enhancer activity in mESCs. In N2A, which express Zmynd8as and Brd1as at low levels relative to undifferentiated mESCs, this enhancer activity is lost. Taken together, we find that enhancer activity at these two loci is limited to mESCs in the three cell types we have tested, and further that expression of Zmynd8as, Brd1as, and the short isoforms of Zmynd8 and Brd1 is strongest in mESCs. These data suggest that Zmynd8as and Brd1as expression, as well as expression of the short isoforms of Zmynd8 and Brd1, is correlated with active enhancer activity at these P300 sites.

### Functional characterization of Zmynd8as and Brd1as

We next focused on characterizing Zmynd8as and Brd1as function. In particular, we sought to uncover the means through which these transcripts might regulate expression of their corresponding sense genes. The finding that short isoforms of Zmynd8 and Brd1 appear to be preferred upon expression of Zmynd8as and Brd1as is suggestive of a *cis*-regulatory mechanism, particularly transcriptional interference, in which the act of transcription of Zmynd8as and Brd1as hinders the elongation of transcription of Zmynd8 and Brd1. However, the fact that Zmynd8as and Brd1as are antisense transcripts, and in particular the fact that processed Zmynd8as overlaps an exon of processed Zmynd8, also suggests this regulation could occur in *trans* through a base-pairing mechanism. We thus sought to distinguish between these two potential general mechanisms.

**Figure 4:**
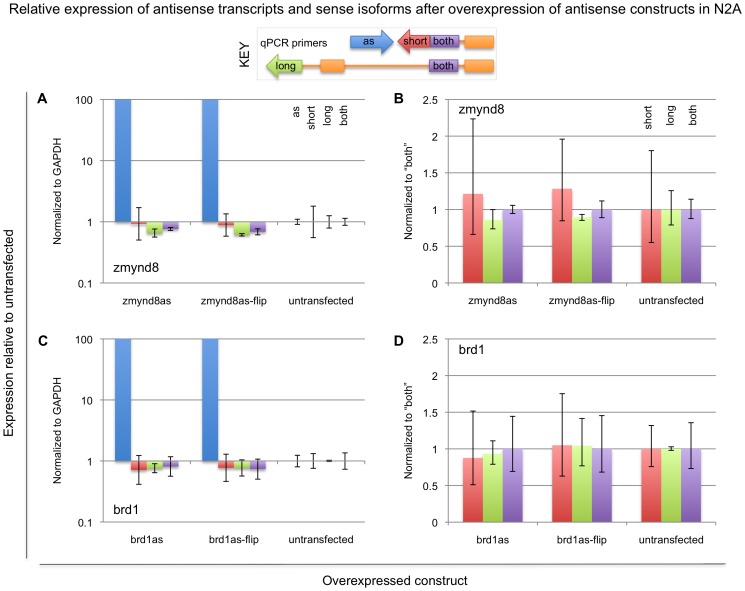
Expression levels of Zmynd8- and Brd1-associated transcripts in N2A cells after overexpression of antisense transcripts. cDNAs for Zmynd8as, Brd1as, or their reverse complements (zmynd8asflip, brd1asflip) were overexpressed from an exogenous plasmid driven by a CMV promoter, and relative expression of their associated transcripts was measured with quantitative RT-PCR, using primers specific to the antisense transcripts (“as”, blue bars), the short sense isoforms (“short”, red bars), the long sense isoforms (“long”, green bars), or both sense isoforms (“both”, purple bars). Chart coloring is also indicated in the key. In each case, more than 10000-fold upregulation was observed for the introduced cDNA; the graphs in (A) and (C) have been clipped. Expression values are reported relative to untransfected N2A, normalized to GAPDH (A and C), Zmynd8-both (B), or Brd1-both (D). Note that the untransfected cells represent a qualitatively different cell type than the transfected cells, but are shown as an additional control.

To determine whether Zmynd8as or Brd1as can act through a *trans* mechanism to regulate Zmynd8 or Brd1, we overexpressed each transcript from a CMV promoter-containing plasmid in N2A, which has quite low endogenous expression of each of these transcripts (Fig. 3A). We collected RNA samples 24 hours after transfection and measured the relative expression of each antisense transcript and its associated sense isoforms with qRT-PCR. We found overexpression of Zmynd8as and Brd1as from an exogenous locus has no effect on the expressed isoforms of Zmynd8 and Brd1 (Fig. 4). These results argue against a *trans* mechanism such as antisense targeting, but are consistent with the idea that Zmynd8as and Brd1as could regulate Zmynd8 and Brd1 isoform expression through a *cis* mechanism such as transcriptional interference. Additionally supporting our notion that Zmynd8as and Brd1as do not function in *trans*, we find exogenous expression of Zmynd8as does not increase activity of the Zmynd8as enhancer (Fig. S13; Text S1).

**Figure 5:**
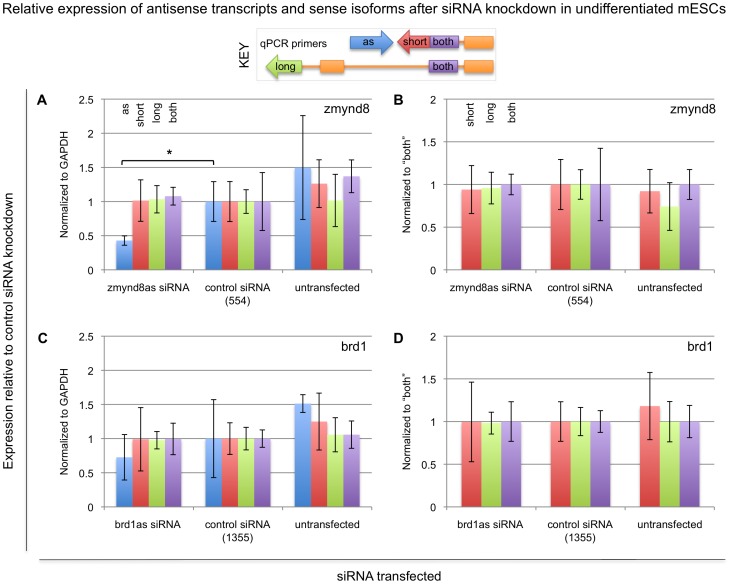
Expression of Zmynd8 and Brd1 isoforms after siRNA knockdown of Zmynd8as and Brd1as in mESCs. siRNAs designed to Zmynd8as and Brd1as as well as control (scrambled) siRNAs were transfected into undifferentiated mESCs and expression of the various associated transcripts was measured with quantitative RT-PCR, using primers specific to the antisense transcripts (“as”, blue bars), the short sense isoforms (“short”, red bars), the long sense isoforms (“long”, green bars), or both sense isoforms (“both”, purple bars). Chart coloring is also indicated in the key. Expression values are reported relative to the levels observed in the mESCs transfected with the control siRNAs, normalized to GAPDH (A and C), Zmynd8-both (B), or Brd1-both (D). Shown in all panels are averages and 95% confidence intervals over three biological replicates. Note that the untransfected cells represent a qualitatively different cell type than the siRNA-transfected cells, but are shown as an additional control. As an alternate measure of statistical significance, p-values for experimental siRNA/control siRNA comparisons are indicated where appropriate. *, p<0.05; two-tailed paired Student's t-test.

We next knocked down expression of Zmynd8as and Brd1as in undifferentiated mESCs (cell line 46C) using siRNAs targeting the 3′ ends of each transcript, in regions not overlapped by processed Zmynd8 or Brd1. siRNAs are designed to participate in the RNAi pathway to achieve knockdown of their targeted RNAs. Since the target cleavage reaction of the RNAi pathway is cytoplasmic, siRNA knockdown of Zmynd8as and Brd1as is expected to occur post-transcriptionally, on fully processed Zmynd8as and Brd1as, in the cytoplasm only. Note siRNAs are double-stranded, but by designing the siRNAs to regions not present in the processed forms of Zmynd8 or Brd1 we expect that only Zmynd8as and Brd1as will be targeted by their respective siRNAs. To further ensure strand specificity, we used custom Stealth RNAi siRNAs (Invitrogen): with this technology the sense siRNA strand cannot enter the RNAi pathway. We transfected these siRNAs into mESCs, collected RNA samples 24 hours after transfection, and measured the relative expression of each antisense transcript and its associated sense isoforms with qRT-PCR. We found we achieved efficient siRNA-mediated knockdown of Zmynd8as, yet this knockdown has no effect on Zmynd8 isoform expression (Fig. 5A,B). Our siRNA-mediated knockdown of Brd1as was less efficient; however, our data suggests Brd1 isoforms are also not subsequently affected (Fig. 5C,D). As with the overexpression experiments, these results are again consistent with a model in which Zmynd8as and Brd1as regulate Zmynd8 and Brd1 isoform expression in the nucleus through a *cis* mechanism such as transcriptional interference.

**Figure 6:**
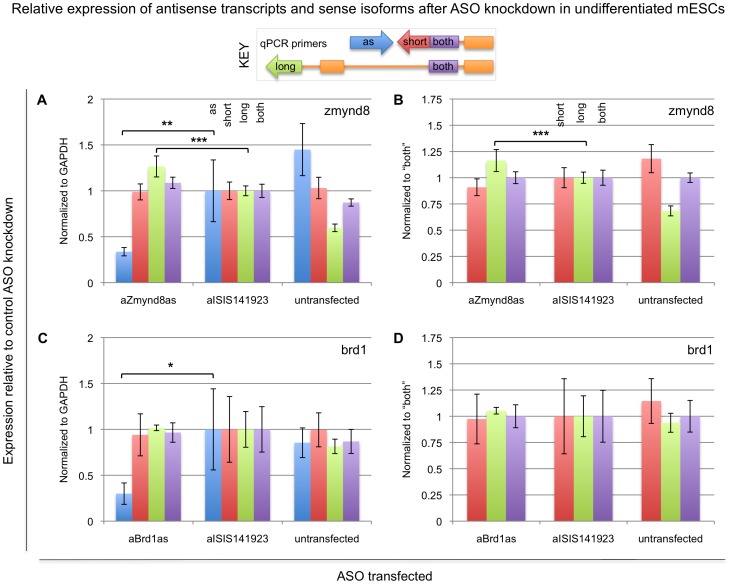
Expression of Zmynd8 and Brd1 isoforms after ASO knockdown of Zmynd8as and Brd1as in mESCs. Knockdown ASOs [35] designed antisense to Zmynd8as and Brd1as were transfected into undifferentiated mESCs, and expression of the various associated transcripts was measured with quantitative RT-PCR, using primers specific to the antisense transcripts (“as”, blue bars), the short sense isoforms (“short”, red bars), the long sense isoforms (“long”, green bars), or both sense isoforms (“both”, purple bars). Chart coloring is also indicated in the key. An additional ASO not matching any sequence in the mouse genome (ISIS141923, [60]) was included as a control. Expression values are reported relative to the levels observed in the mESCs transfected with the control ASOs, normalized to GAPDH (A and C), Zmynd8-both (B), or Brd1-both (D). Shown in all panels are averages and 95% confidence intervals over three biological replicates; (A) and (B) show the combined results of two separate experiments, each with three biological replicates. Note that the untransfected cells represent a qualitatively different cell type than the ASO-transfected cells, but are shown as an additional control. As an alternate measure of statistical significance, p-values for experimental ASO/control ASO comparisons are indicated where appropriate. *, p<0.05; **, p<0.01; ***, p<0.001; two-tailed paired Student's t-test.

As a final test of Zmynd8as and Brd1as function, we used the antisense oligo (ASO) method most recently described by Ideue et al. [35] to knock down expression of Zmynd8as and Brd1as in undifferentiated mESCs. This method is designed to knock down RNAs via RNaseH, an enzyme localized to the nucleus of all cells, with cleavage activity on the RNA strand of an RNA:DNA hybrid. This method has been demonstrated to reliably knock down nuclear RNAs that evade destruction by siRNA methods. Further, since this method uses a single-stranded oligo, it is clearly strand-specific. In each case we designed ASOs in the same regions as the siRNAs used previously. As before, we transfected mESCs with the ASOs, collected RNA samples 24 hours after transfection, and measured expression of the various transcripts using qRT-PCR. As shown in Figure 6, the ASOs achieved efficient knockdown of both Zmynd8as and Brd1as. Interestingly, our results suggest that nuclear knockdown of Zmynd8as results in an increased relative amount of the Zmynd8 long isoform, partially recapitulating the differences in relative endogenous expression levels observed in NP and N2A versus mESCs (compare Fig. 6A,B with Fig. 3A). These results indicate that it is not solely the act of transcription of Zmynd8as that regulates Zmynd8 expression, but rather that the Zmynd8as transcript itself may play a role in the regulation of Zmynd8. Further, the regulatory activity of Zmynd8as must occur in the nucleus, since cytoplasmic knockdown of Zmynd8as does not show this effect (Fig. 5). Conversely, nuclear knockdown of Brd1as shows no effect on Brd1 isoforms (Fig. 6C,D). This finding is consistent with Brd1as regulating Brd1 through a simple transcriptional interference model.

The fact that Zmynd8as knockdown shows effects on gene expression, yet Zmynd8as overexpression does not, is perhaps surprising but is supported by previous literature. This phenotype is seen for the recently characterized HOTTIP RNA [36], and also for the RNAs with enhancer-like activity described by Orom et al. [37]; note that in each of these cases siRNAs only were used to achieve knockdown.

## Discussion

### Hypotheses for enhancer-associated antisense ncRNA action

**Figure 7:**
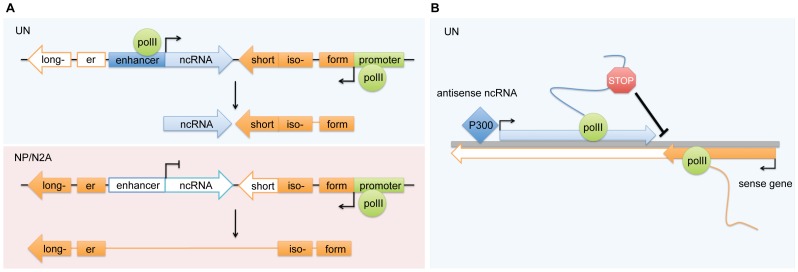
Proposed models for gene isoform specificity through enhancer-associated antisense transcription. (A) Cartoon representation of Zmynd8as and Brd1as expression and enhancer activity. In each case, in undifferentiated mESCs (UN) the antisense ncRNA is expressed (blue block arrow), there is P300 binding towards the 5′ end of the ncRNA, and the associated sense protein-coding gene (orange block arrow) prefers a short isoform. In NP and N2A, the antisense ncRNA is not expressed, P300 binding is lost, and the associated sense protein-coding gene prefers a longer isoform. We propose that enhancer-driven transcription of the antisense ncRNAs facilitates short sense isoform selection in *cis*. In the case of Brd1as, this *cis* regulation appears to occur through a basic transcriptional interference model, in which transcription of Brd1as hinders the elongation of Brd1 transcription. (B) Proposed model for Zmynd8as action. The nascent Zmynd8as transcript, driven by p300 binding at its 5′ region and tethered to the chromosome by pol II, elicits an as-yet-unknown stop signal that stalls extension of the pol II transcribing Zmynd8. The stop signal may be an inhibitory factor binding to the nascent Zmynd8as transcript that is thus localized to the 3′ region of Zmynd8-short; alternately, the stop signal could be RNA processing machinery or other transcription-associated factors that pose additional steric hindrance for any pol II units transcribing Zmynd8. Stalling of the Zmynd8 pol II may facilitate transfer of the polyadenylation machinery from the nascent Zmynd8as transcript to the nascent Zmynd8 transcript, thus resulting in the shorter Zmynd8 isoform. Block arrows represent the relative genomic locations of full length Zmynd8as and Zmynd8.

As detailed above, expression of Zmynd8as and Brd1as is correlated with active enhancer activity at P300 sites at the 5′ ends of each of these transcripts. In both NPs and N2A cells, Zmynd8as and Brd1as expression is sharply decreased relative to mESCs. Further, in these two cell types enhancer activity at the mESC P300 sites is abolished, as determined by the lack of p300 binding in NPs as well as the inability of these regions to significantly upregulate luciferase reporter expression in N2A and NPs. Expression of Zmynd8as and Brd1as additionally correlates with increased frequency of Zmynd8 and Brd1 taking short isoforms. We therefore suggest that enhancer activity at Zmynd8as and Brd1as drives expression of these transcripts, and in this manner the enhancers are able to control isoform specificity of the coding genes Zmynd8 and Brd1 (Fig. 7A). Note that the phenomenon of transcription originating from active enhancers is well established (see most recently [8], [9]), yet new work has demonstrated some promoters also can exhibit enhancer activity [38], blurring the distinctions between enhancers and promoters. Thus while it is clear that the Zmynd8as and Brd1as P300 sites exhibit enhancer activity (Fig. 3C), the possibility remains that these regions are promoters with enhancer function, rather than strict enhancers that originate transcripts.

The exact manner in which Zmynd8as and Brd1as control isoform specificity of Zmynd8 and Brd1 is a matter requiring further study. In the case of Brd1as, this regulation appears to occur simply through transcriptional interference. In the case of Zmynd8as, knockdown data suggest that nuclear posttranscriptional downregulation of Zmynd8as increases expression of the long isoform of Zmynd8; this finding suggests that it is not solely the act of transcription of Zmynd8as that regulates Zmynd8 isoform selection. Further, this regulation must be accomplished by nuclear Zmynd8as, as knockdown of cytoplasmic Zmynd8as via siRNAs does not lead to the same upregulation of Zmynd8-long. Curiously, and similarly to HOTTIP [36] and the enhancer RNAs desribed by Orom et al. [37], exogenous expression of Zmynd8as shows no effect on Zmynd8 expression, suggesting Zmynd8as has strictly local regulatory capabilities.

With these points in mind, we propose that Zmynd8as regulates Zmynd8 transcription while tethered to the chromosome at its site of origin, in a manner similar to the CCND1 upstream ncRNAs [39]. Since it is clear from our RNA-Seq data that Zmynd8as does not strictly remain in the nucleus, an obvious candidate for this tethering is simply the pol II unit transcribing Zmynd8as. The nascent Zmynd8as transcript then elicits an undetermined stop signal that stalls the extension of pol II molecules transcribing Zmynd8 (Fig. 7B), and potentially facilitates the transfer of the polyadenylation machinery from the nascent Zmynd8as transcript to the nascent Zmynd8 transcript. In this model, knockdown with ASOs [35] cleaves the nascent Zmynd8as transcript from the pol II tether, thus removing the undetermined stop signal and allowing the pol II molecule transcribing Zmynd8 to continue unimpeded. Alternately, the ASO may interfere with the binding of any inhibitory factors to nascent Zmynd8as, and thus prevent localization of the undetermined stop signal.

Other factors associated with the nascent antisense transcript could contribute to the stop signal. For example, it is likely that splicing and polyadenylation of Zmynd8as occurs co-transcriptionally (for reviews of co-transcriptional RNA processing, see [40], [41]). Thus, spliceosomes and other processing factors bound to nascent Zmynd8as may serve as additional roadblocks hindering further extension of pol II units transcribing Zmynd8. This model could help explain the differences observed between Zmynd8as and Brd1as (which lacks introns), the expression of Zmynd8 and Brd1 isoforms, and their knockdown phenotypes. Clearly the relative ratio of short versus long isoforms is greater in the case of Zmynd8/Zmynd8as than in the case of Brd1/Brd1as. As Brd1as is unspliced, its transcribing unit (pol II plus nascent Brd1as transcript) may pose a smaller obstacle towards opposing (sense) pol II units than does the Zmynd8as transcribing unit (pol II plus nascent Zmynd8as transcript plus spliceosomes and/or other RNA processing factors). This final model is a refinement of the transcriptional interference model in which it is not simply the transcriptional machinery, but also RNA processing machinery, that interferes with transcription of the negatively targeted gene.

### Indications of a more widespread mechanism

Regardless of the exact mechanism through which Zmynd8as and Brd1as regulate expression and despite the fact that neither is highly conserved, the fact that both Zmynd8 and Brd1 appear to be controlled by enhancer-driven antisense transcription suggests this may be a common mechanism for gene regulation. Curiously, Zmynd8 and Brd1 are functionally related proteins. Zmynd8 has been implicated in chromatin silencing and transcriptional repression [42], [43], particularly of neuronal genes in non-neuronal lineages [43]. Brd1 is reported to act in a complex with HBO1, a MYST histone acetyltransferase, to establish acetylation of histone 3 lysine 14 (H3K14Ac) at developmental regulator genes [44]. But perhaps more tellingly, Zmynd8 and Brd1 share many functional protein domains, each containing zinc finger, bromodomain, and PWWP domains [29], [30] (Fig. 22; Text S1). In the mouse genome, there are a total of 1410 proteins containing zinc finger domains, 39 containing bromodomains, and 21 containing the PWWP domain. Five mouse proteins contain all three types of domains, including Zmynd11, Brpf1, and Brpf3; these same 5 proteins also comprise the set of all mouse proteins that contain both a bromodomain and the PWWP domain. Nineteen mouse proteins contain both zinc finger and bromodomains. We did not detect internal antisense transcription for any of these related genes in our samples, but such transcription could possibly occur in other cell types.

Further supporting the idea that enhancer-associated antisense transcription could be prevalent in other cell types, Zmynd8as and Brd1as do not show sequence homology per BLAST [31], despite the fact that Zmynd8 and Brd1 are functionally related. This finding indicates that these antisense transcripts have evolved independently of each other. Notably, Brd1as originates from an ORR1A2 MaLR LTR, a family of elements present only in rodents [45] (Fig. S12). Zmynd8as also is largely murine-specific, though it does not appear to have arisen from an LTR. The murine-specificity of these two transcripts hints that enhancer-driven antisense transcription could also be a means for species- or lineage-specific gene isoform regulation. With the wealth of RNA-Seq and ChIP-Seq data now emerging, it will be interesting to see if more cases such as these can be found in other tissues and organisms. A limitation of most RNA-Seq data to date is that it is not strand-specific. Indeed, Guttman et al. recently observed Brd1as in their mESC RNA-Seq data, but the lack of strand-specificity in their library generation protocol prompted them to erroneously merge the short isoform of Brd1 with what is in fact the Brd1as transcript [46]. RNA-Seq technology is constantly improving, however, and more options for strand-specific library generation exist now than ever before, providing hope the necessary data will surface in the quite near future.

### Potential consequences of the short sense isoforms specified through enhancer-associated antisense transcription

Another remaining question concerns the function of the short isoforms of Zmynd8 and Brd1. The short Zmynd8 protein will lack the MYND functional domain present in the full-length Zmynd8 protein (Fig. S14; Text S1). It is expected that the loss of the MYND domain would compromise functionality of the short Zmynd8 protein. Indeed, the MYND domain is necessary for Zmynd8's interaction with RCOR2 to establish transcriptional repression [43], [47]. Similarly, the short Brd1 protein must lack the PWWP domain contained in full-length Brd1 (Fig. S14; Text S1). As stated earlier, Brd1 acts in a complex with HBO1 to establish histone acetylation [44]. This interaction occurs through the 198 N-terminal residues of Brd1 [44], which are preserved in Brd1-short. However, the loss of the PWWP domain, which is capable of binding DNA and methyllysine histones [48], may inhibit the proper localization or targeting of Brd1-short and any complexes it may form [49]. Thus the net result of enhancer-driven Zmynd8as and Brd1as expression may be expected to be negative regulation of Zmynd8 and Brd1 function. This conclusion is striking in that enhancers have traditionally been considered to effect only positive regulation, strictly at promoters. An interesting but unexplored related hypothesis is that in mESCs, the P300-marked enhancer sites at Zmynd8as and Brd1as may target the Zmynd8 and Brd1 promoters to additionally drive transcription of the sense Zmynd8 and Brd1 genes. In this case, the enhancers would “loop around” to interact with the Zmynd8 and Brd1 promoters. Such an interaction would additionally bring the Zmynd8as and Brd1as loci in close proximity to the enhancer/sense gene promoter locus, potentially facilitating transcription of Zmynd8as and Brd1as as pol II is recruited to the locus. Zmynd8as and Brd1as expression in this case may act as negative feedback to keep full-length Zmynd8 and Brd1 expression at the correct levels. Experiments examining chromatin structure, such as chromosome conformation capture (3C; [50]), could undoubtedly shed light on this hypothesis.

There are only limited previously described cases of coordinated enhancer:ncRNA activity. In what is perhaps the best known example, the case of ncRNA Evf-2 and its associated enhancer, the ncRNA acts in the complex that binds the enhancer to drive transcription of target genes Dlx5 and Dlx6 [13]. The mode of enhancer:ncRNA interaction that we have presented here differs greatly, suggesting that there is still much to learn about enhancer:ncRNA cooperation, and that many other types of functional interactions may yet be discovered. Indeed, much progress has been made, but still we are just beginning to understand the many complexities of enhancer:ncRNA interactions, and of enhancer function in general.

## Materials and Methods

### Accession codes

The RNA-Seq data discussed in this publication have been deposited in NCBI's Gene Expression Omnibus [51] and are accessible through GEO Series accession number GSE38990 (http://www.ncbi.nlm.nih.gov/geo/query/acc.cgi?acc=GSE38990). See Text S1 for details about RNA-Seq library construction, read mapping, and library assessment.

### Cell culture

mESCs (cell line 46C [52]) were cultured without feeder layers according the BayGenomics protocol adapted from [53]. mESCs were differentiated to Day 5 NPs with the protocol described by Ying et al. [54], [55]. Note an alternate neural differentiation protocol was used for ChIP-qPCR [34]; see ChIP-qPCR section in Methods. N2A (Neuro-2A, ATCC) cells were cultured in Dulbecco's Modified Eagle Medium with 10% FBS and NEAA. All cell types were transfected with Lipofectamine 2000 (Invitrogen), according to the manufacturer's instructions.

### RACE and cloning of full-length transcripts

5′ and 3′ RACE was performed on total RNAs from mESCs and Day 5 NPs (nuclear and cytoplasmic fractions as described previously [14]; see also Text S1) through the GeneRacer (Invitrogen) system, using gene-specific primers as indicated in Table S4. Primer names indicate whether a primer was used in 5′ or 3′ RACE. Based on RACE products, full-length cDNAs for Zmynd8as and Brd1as were cloned into the Invitrogen Gateway system (pENTR-D/TOPO); forward and reverse cloning primers are also listed in Table 4 with “Cdna” primer names. Note forward cDNA primers have “CACC” sequence at their 5′ ends to enable directional cloning. All cDNA clones were sequence-verified against the mm9 assembly of the mouse genome on the UCSC Genome Browser; mismatches relative to the reference genome were permitted if represented in dbSNP [56]. For expression experiments, cDNA clones were moved to the pcDNA 3.2/V5-DEST (Invitrogen) vector using the Gateway LR Cloning system (Invitrogen).

### ChIP-qPCR

mESCs (cell line 46C) were differentiated to Day 6 and Day 10 neural precursors as described by Eiraku et al. [34]. Chromatin immunoprecipitation of undifferentiated mESCs, Day 6 neural precursors, and Day 10 neural precursors was performed as described by Li et al. [57] using p300 (C20) X (Santa Cruz Biotechnology) and Normal Rabbit IgG antibodies (Santa Cruz Biotechnology), followed by amplification of ChIP-selected DNA using the Sigma Whole Genome Amplification Kit, according to the manufacturer's instructions. 20 ng of each sample was then reamplified again using the Whole Genome Amplification Kit for use in ChIP-qPCR.

qPCR was performed with iQ SYBR Green Supermix (Biorad) using 5 ng of ChIP DNA per reaction; all reactions were set up in triplicate. Cycling was performed on a Rotor-Gene 6000 (Qiagen) according to the iQ SYBR Green Supermix instructions. Primers used for ChIP-qPCR are indicated in Table S4.

### Luciferase assay

Regions spanning the p300 sites of Zmynd8as and Brd1as were cloned into pENTR-D/TOPO (Invitrogen), with primers indicated below. All clones were sequence-verified against the mm9 assembly of the mouse genome on the UCSC Genome Browser; mismatches relative to the reference genome were permitted if represented in dbSNP [56].

Enhancer clones were introduced into a pGL4.12 vector (Promega) modified to include a thymidine kinase (TK) promoter upstream of firefly luciferase. This TK-pGL4.12 vector was a gift from the laboratory of Dr. Martin Privalsky at the University of California, Davis. For downstream enhancer tests, a Gateway rfB cassette was introduced into the TK-pGL4.12 vector at the BamHI site, downstream of the SV40 late poly(A) signal, using the Gateway Vector Conversion System (Invitrogen), and enhancer clones were introduced into this vector from pENTR-D/TOPO using the Gateway LR system (Invitrogen). Cells were transfected in triplicate with a 9∶1 mass ratio of enhancer construct:pRL-TK renilla luciferase control plasmid (Promega) using Lipofectamine 2000 (Invitrogen) according to the manufacturer's instructions. Cells were assayed approximately 24 hours later with the Dual-Luciferase System (Promega), and luciferase activity was measured in a Victor Light Luminescence Counter (Perkin-Elmer); three technical replicates were measured for each biological replicate. In figures, luciferase activity (ratio of firefly counts per second/renilla counts per second) for each enhancer construct is shown relative to the activity observed for TK-pGL4.12 (ratio of enhancer construct luciferase activity: TK-pGL4.12 activity). Error bars represent 95% confidence intervals over the three biological replicates. All experiments were repeated at least twice. See Text 8 for luciferase assays presented in Figure S13.

### qRT-PCR

qRT-PCR was performed using the QuantiTect SYBR Green RTPCR Kit (Qiagen), using 100 ng of total RNA per reaction; all reactions were set up in triplicate. Cycling was performed on a Rotor-Gene 6000 (Qiagen) according to the QuantiTect SYBR Green RTPCR Kit instructions. Primers used for qRT-PCR are indicated in Table S4.

### Overexpression

Sequence-verified entry clones for Zmynd8as, Brd1as, and their reverse complements were introduced into the mammalian expression vector pcDNA3.2-V5/DEST (Invitrogen) through the Gateway system (Invitrogen); this vector uses a CMV promoter to drive expression of its inserts. Constructs were transfected into N2A with Lipofectamine 2000 (Invitrogen), according to the manufacturer's instructions. RNA was harvested 24 hours after transfection for qRT-PCR experiments using Trizol reagent (Invitrogen). RNA extraction was followed by DNase treatment and cleanup with RNeasy columns (Invitrogen) to ensure removal of plasmid DNA.

### Knockdown

Stealth siRNAs and controls were designed with the Invitrogen BLOCK-iT RNAi designer and ordered from Invitrogen. ASOs were ordered from Integrated DNA Technologies (IDT) with sequences indicated in Table S4.

siRNAs and ASOs were transfected into undifferentiated mESCs with Lipofectamine 2000 (Invitrogen) according to the Stealth RNAi transfection protocol provided with the reagent. RNA was harvested from cells 24 hours after transfection for use in qRT-PCR experiments using Trizol reagent (Invitrogen).

## Supporting Information

Text S1 **Supporting Methods and References.** Methods for neural differentiation, RNA-Seq library construction, RNA-Seq data analysis, transcript assembly, and characterization of Zmynd8as, Zmynd8, and Brd1. Also contains Supporting References 65–85.
(PDF)

Figure S1 **Schematic for intial mapping of RNA-Seq libraries.** Mapping was performed in stages with the SOLiD Corona pipeline; see Text S1.
(TIF)

Figure S2
**Coverage of known cytoplasmic and nuclear RNAs in RNA-Seq libraries.** (A) RNA-Seq coverage tracks for 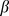-Actin, a known cytoplasmic RNA. In both libraries, coverage in exons greatly exceeds coverage in introns, but the nuclear library has greater intronic coverage than the cytoplasmic library. (B) Coverage tracks for AIR, a ncRNA known to be nuclear-retained and to evade splicing [61]. Coverage track heights (in number of reads) are indicated to the immediate left of each coverage track and are scaled according to the number of reads mapped for each RNA-Seq library. Pink coloring at the top of a coverage track indicates the number of reads mapping at that particular location exceeds the range of the track. Note track heights for panel (A) were chosen to highlight the intronic coverage observed in the nuclear library. See Text S1.(TIF)

Figure S3 **Coverage of known housekeeping genes in RNA-Seq libraries.** Distributions of RPKM values for each expression level category defined by Warrington et al. [16] are shown for each RNA-Seq library type. L, low, consisting of 11 genes; LM, low-medium, 89 genes; M, medium, 230 genes (2 genes with RPKM values of 0 in all library types omitted); MH, medium-high, 22 genes; H, high, 15 genes. Data is presented in modified boxplot format. Lower and upper boundaries of boxes represent data values at the first and third quartiles, respectively. Inner bold lines indicate median data points. Whiskers extend no more than 1.5 times the interquartile distance from the first and third quartiles and represent the lowest and highest data points within this range, respectively. All other data points are plotted as outliers with open circles. See Text S1.
(TIF)

Figure S4 **Comparison of neural differentiation protocols.** Comparision of neural differentiation protocols used in Abranches et al. 2009 [17] (A), and this study (B). Image modified from [17]. See Text S1.
(TIF)

Figure S5 **NP specificity distributions for Abranches et al. gene sets.** As a measure of tissue specificity, the NP specificity scores for the genes reported upregulated in each cell type by Abranches et al. [17] are shown. See Text S1 for discussion of NP specificity. Modified boxplots are shown as in Figure S3.
(TIF)

Figure S6 **Differences in expression values for adjacent internal vs. adjacent outer exons.** Expression value differences are shown as the absolute value of the RPKM percentile differences between the two exons. “in,” internal; “out,” outer. Modified boxplots are shown as in Figure S3. See Text S1.
(TIF)

Figure S7 **Distribution of RPKM percentile differences (averages per gene) in adjacent internal exons.** The 90th, 95th, and 99th percentile values are indicated for each RNA type. See Text S1.
(TIF)

Figure S8 **Distribution of RPKM percentile differences in adjacent outer exons.** The 90th, 95th, and 99th percentile values for the adjacent internal exons shown in Figure S7 are plotted over top; the percentage of adjacent outer exons with values less than or equal to these values are indicated. See Text S1.
(TIF)

Figure S9 **Number of fragments per UCSC Known Gene before and after merging Cufflinks transcripts.** “CL,” Cufflinks transcripts with no merging; “M5,” merged Cufflinks transcripts allowing an RPKM percentile as great as 5; “M10,” merged Cufflinks transcripts allowing an RPKM percentile difference as great as 10. In merged cases, an “intron length” of up to 11 kb was allowed. Modified boxplots are shown as in Figure S3. See Text S1.
(TIF)

Figure S10 **Merged Cufflinks transcripts at Nestin in undifferentiated nuclear library.** RNA-Seq read coverage for Nestin is shown in the first track, above UCSC Genes, second track. Cufflinks (v0.8.1) output is third track from top; note Cufflinks predicts several fragmented transcripts along Nestin. Fourth track from top, merged Cufflinks output allowing an RPKM percentile difference of 10 and an “intron length” of 11 kb; sixth track from top, merged Cufflinks output allowing an RPKM percentile difference of 5 and an “intron length” of 11 kb. See Text S1.
(TIF)

Figure S11 **Zmynd8as and Zmynd8-short transcript structures.** Structures determined with 5′ and 3′ RACE [26], [27] on undifferentiated mESC nuclear and cytoplasmic RNA; RACE products are indicated in the teal “RACE” track. Note that the apparent final splice site in the zmynd8-3race product does not represent a true intron, but a structural difference (gap) in the 46C genome relative to the reference mouse genome; see Text S1. Immediately below are shown cDNA and enhancer clones generated for Zmynd8as experiments (“Clones” track). Repeat elements are shown in the RepeatMasker track [62], second from bottom. All other tracks are as in Figure 2 of the main text: blue coverage tracks indicate the number of uniquely placed unspliced reads mapped per base on the positive strand; orange coverage tracks indicate the same for the negative strand. Coverage track heights (in number of reads) are indicated to the immediate left of each coverage track and are scaled according to the number of reads mapped for each RNA-Seq library. Also shown are UCSC Known Genes [28], along with P300 binding data (ChIP-Seq peaks) from undifferentiated mESCs [19], H3K4Me1 data (ChIP-Seq peaks) from mESCs and NPs [23], mammalian conservation (PhastCons) [58], [59], and alignments against several other species (Multiz) [63]. Known gene Zmynd8 is annotated on the negative strand, with both short and long isoforms reported; positive strand (antisense) transcription is seen in undifferentiated cytoplasmic and undifferentiated nuclear RNA.
(TIF)

Figure S12 **Brd1as and Brd1-short transcript structures.** Structures determined with 5′ and 3′ RACE [26], [27] on undifferentiated mESC nuclear and cytoplasmic RNA; RACE products are indicated in the teal “RACE” track. Immediately below are shown cDNA and enhancer clones generated for Brd1as experiments (“Clones” track). Repeat elements are shown in the RepeatMasker track [62] second from bottom; interestingly Brd1as originates from a MaLR LTR. All other tracks are as in Figure 2 of the main text: blue coverage tracks indicate the number of uniquely placed unspliced reads mapped per base on the positive strand; orange coverage tracks indicate the same for the negative strand. Coverage track heights (in number of reads) are indicated to the immediate left of each coverage track and are scaled according to the number of reads mapped for each RNA-Seq library. Also shown are UCSC Known Genes [28], along with P300 binding data (ChIP-Seq peaks) from undifferentiated mESCs [19], H3K4Me1 data (ChIP-Seq peaks) from mESCs and NPs [23], mammalian conservation (PhastCons) [58], [59], and alignments against several other species (Multiz) [63]. Known gene Brd1 is annotated on the negative strand, with a novel 3′ end expressed in undifferentiated cells confirmed by RACE; positive strand (antisense) transcription is seen in undifferentiated cytoplasmic and undifferentiated nuclear RNA.
(TIF)

Figure S13 **Test of enhancer activity in mESCs and NP cells with Zmynd8as cDNA.** Enhancer constructs, with the Zmynd8as P300 site cloned upstream of the TK promoter, were transfected into undifferentiated mESCs (A) or NP neural precursors (B) along with a plasmid driving either Zmynd8as, its reverse complement, or GFP under a CMV promoter. TK, promoter only luciferase reporter vector; enhTK and enhflipTK, Zmynd8as P300 site cloned upstream of the TK promoter and luciferase reporter, in forward and reverse orientations, respectively; GFP, GFP plasmid under control of a CMV promoter; cDNA and cDNAflip, plasmids containing Zmynd8as and its reverse complement under the control of a CMV promoter.
(TIF)

Figure S14 **Functional domains in long and short isoforms of Zmynd8 and Brd1.** Both long and short isoforms of Zmynd8 contain a zinc finger domain (ZNF; representing RING/FYVE/PHD-type domains), a bromodomain (Brom), a PWWP domain (PW), and a domain of unknown function DUF3544 (DUF); the long form also contains a zinc finger MYND-type domain (MYND). Both long and short isoforms of Brd1 contain an enhancer of polycomb-like, N-terminal domain (EPL) and two zinc finger domains and a bromodomain, as in Zmynd8; the long form also contains a PWWP domain. Numbers at bottom indicate lengths of the isoforms in amino acids. Domains determined with InterProScan [29], [30].
(TIF)

Table S1 **RNA-Seq mapping statistics.** Mapping statistics for reads from RNA-Seq experiment. Sample, RNA library information; Barcode, barcode used for sequencing; Count, total number of reads returned; Filtered, percent of reads passing filter step; Mapped, percent of reads mapped singly or multiply; Unique, percent of reads mapped uniquely. Barcodes B14–B15 were part of a separate project and were not used in this study, but are presented here for completeness of run information. “UN” indicates our undifferentiated mESC libraries, while “D5” indicates our day 5 neural precursor libraries.
(PDF)

Table S2 **Bad joins of UCSC Known Genes in Cufflinks and merged Cufflinks output.** “Bad joins” occur when two separate genes are merged into a single transcript. Cufflinks bad joins, bad joins by Cufflinks; d10, bad joins when an RPKM percentile difference of 10 is allowed for merging Cufflinks output; d5, bad joins when an RPKM percentile difference of 5 is allowed for merging Cufflinks output.
(PDF)

Table S3 **ncRNA candidates for study from the RNA-Seq project.** Columns indicate the following information: Region, genomic coordinates for area of novel transcription on the UCSC Genome Browser mouse assembly mm9; Strand, strand of novel transcription; Nearest Gene, nearest known gene; Description, properties of novel transcription; Expression, RNA-Seq libraries containing the novel transcription; Enhancer, cell types with enhancer activity in this area, with enhancers defined from previous literature as stated in the text of this section. Two identified regions correspond to lincRNAs first reported by Guttman et al. [64]. NP, neural precursors.
(PDF)

Table S4 **Sequences of primers, siRNAs, and ASOs used in this study.** Primers were used for a variety of purposes, indicated in the “Purpose” column. For RACE primers, primer names indicate whether the primer was used in 5′ or 3′ RACE. Note forward cloning primers have “CACC” at their 5′ ends to allow directional cloning with the Invitrogen Gateway System. For ASOs, the five terminal nucleotides on each end are 2′-Omethoxyethyl nucleotides indicated as “mN,” where N is the nucleotide. The phosphothioate backbones are indicated with asterisks.
(PDF)
