# Supplementary material for: Gene Isoform Specificity through Enhancer-Associated Antisense Transcription
Source: PLoS One. 2012 Aug 24;7(8):e43511. doi: 10.1371/journal.pone.0043511 (PMC3427357; doi:10.1371/journal.pone.0043511)
Supplement: Text S1 — Supporting Methods and References. Methods for neural differentiation, RNA-Seq library construction, RNA-Seq data analysis, transcript assembly, and characterization of Zmynd8as, Zmynd8, and Brd1. Also contains Supporting References 65–85. (PDF) [file pone.0043511.s001.pdf]

Text S1. Supporting Methods

Gene isoform specificity through enhancer-associated antisense transcription

Courtney S. Onodera[1,5], Jason G. Underwood[1,2,3,6], Sol Katzman[1,2], Frank Jacobs[1,2], David Greenberg[1,4], Sofie R. Salama[1,2,3,*], David Haussler[1,2,3]

1 Department of Biomolecular Engineering, University of California Santa Cruz, Santa Cruz, CA, United States of America
2 Center for Biomolecular Sciences and Engineering, University of California Santa Cruz, Santa Cruz, CA, United States of America
3 Howard Hughes Medical Institute, University of California Santa Cruz, Santa Cruz, CA, United States of America
4 Department of Molecular, Cell and Developmental Biology, University of California Santa Cruz, Santa Cruz, CA United States of America

∗ **E-mail: ssalama@soe.ucsc.edu**

5 Present address: Institute for Human Genetics, University of California San Francisco, San Francisco, CA United States of America
6 Present address: Pacific Biosciences, Menlo Park, CA, United States of America

Contents

Supporting Methods **2**

Neural differentiation of mESCs 2

Library preparation and SOLiD sequencing 2

Initial mapping of RNA-Seq reads 3

Coverage of known nuclear and cytoplasmic RNAs 4

Coverage of housekeeping genes 4

Comparison with neural differentiation microarray data 5

Novel transcript assembly 6

Overexpression of Zmynd8as does not affect enhancer activity 8

Zmynd8 and Brd1 functional domains and the impacts of the short isoforms 9

References **11**

Supporting Methods

Neural differentiation of mESCs

We followed the protocol of Ying et al. [65,66] to differentiate mESCs to neural precursors (NPs). Briefly, undifferentiated ES cells are plated as a monolayer on gelatin-coated dishes in media lacking leukemia inhibitory factor (LIF), serum, and any growth factors; the absence of LIF prompts mESC differentiation, and in conditions without serum or growth factors the cells largely commit to the neural lineage. The cells acquire a NP morphology by 5 days after plating, marked by expression of SOX1 [66]. We used the 46C cell line, which is a heterozygous knock-in mESC line modified to express green fluorescent protein (GFP) from the SOX1 locus [67]. SOX1 expression is restricted to proliferating neuroectodermal precursors and the lens; thus in this protocol GFP expression can be used to distinguish NPs from any undifferentiated cells which may persist. We performed FACS analysis of NP populations generated through this protocol and found approximately 80% of cells were GFP-positive at day 5 (data not shown). Cells were used at passage number no greater than 10.

Library preparation and SOLiD sequencing

As illustrated in Figure 1 in the main text, cells from each population (undifferentiated, UN, and Day 5 neural precursors, NP) were fractionated to their nuclear and cytoplasmic components using a gentle detergent to disrupt the outer cell membrane while preserving intact nuclei (described in [68]) before RNA extraction. Extracted RNAs (approximately 100-600 μg) were treated with DNase to remove contaminating DNA, then polyadenylated transcripts were selected with Oligotex resin (Invitrogen). We then chose to remove the polyA tails to allow sequencing up to the very 3' ends of the transcripts; to do this we annealed an oligo dT_{18} primer (Invitrogen) to the RNA samples, then treated with RNase H (New England Biolabs). We purified these RNAs using a G50 column (Roche) to remove oligos smaller than 25 bp, followed with another DNase treatment, and then used the Ribominus kit (Invitrogen) to deplete our RNAs further of rRNA. Next, RNAs (400 ng) were fragmented using either 1 μg Nuclease P1 (Sigma) for 10 or 60 minutes as previously described [68], or 1 U RNase III (Applied Biosystems, now Life Technologies) for 10 minutes, according to the instructions in the SOLiD Whole Transcriptome Analysis Kit (Applied Biosystems). The P1 endonuclease, from *Penicillium citrinum*, is known to cut RNA in single-stranded form, whereas RNase III demonstrates more uniform cleavage. It should be noted

that as Nuclease P1 does not fragment uniformly along an RNA molecule, our sequencing reads from this treatment are not expected to be distributed uniformly along transcripts.

We used the Small RNA Expression Kit (SREK, Applied Biosystems) along with our fragmented RNAs to produce the final 11 strand-specific sequencing libraries (Fig. 1, Table S1), with insert sizes of approximately 90-190 bp. Equimolar concentrations of each library (excepting Barcode B06, for which we used twice the concentration) were used as the starting material for emulsion PCR. Our RNA-Seq libraries were sequenced with the SOLiD V3 platform, yielding 50-bp singleton reads. For transcript discovery purposes we disregarded fragmentation enzyme and combined all subsequent sequencing reads from the same cell/compartment type together in our analyses. In the remainder of this supplement, as in the main text, we will use the notations UnNuc, UnCyt, NPNuc, and NPCyt to refer to undifferentiated nuclear, undifferentiated cytoplasmic, day 5 neural precursor nuclear, and day 5 neural precursor cytoplasmic RNAs/libraries/reads, respectively.

Initial mapping of RNA-Seq reads

Reads were mapped in sequential stages to the mouse genome (UCSC assembly mm9) using the Applied Biosystems SOLiD Small RNA Analysis pipeline (Corona), as described previously [68]. First, we generated a filter file that included SOLiD adaptor sequence, rRNAs, and small RNAs (including tRNAs, miRNAs, and others). We also generated a target file (exon+introns) based on UCSC Known Genes [69]: this file contains for each gene the complete genomic sequence from transcription start to transcription end; overlapping genes were merged. Finally, we generated a file containing known exon junction sequences; this file contains 50 bases upstream and downstream of all unique splices represented in UCSC Known Genes. To map reads, we first removed any reads mapping to the filter file, then mapped to the exon+introns target file (sense and antisense). Any remaining reads were then mapped to the genome. We then performed a second round of mapping, first filtering the reads as we did in the first round, and then mapping to the splice junction file. This mapping strategy is illustrated in Figure S1.

At each mapping stage, up to 15 hits per read were allowed. Any reads mapping more than 15 times were discarded. Mapping to the genome was performed chromosome by chromosome, and thus up to 15 hits per chromsome were allowed. In subsequent analyses we have worked only with reads that have mapped singly in our pipeline. Mapping statistics are shown in Table S1. Note that two barcodes, B14 and B15, were part of a separate project and were not used in this analysis, but are presented here for

completeness of run information.

We note that to detect spliced reads, several RNA-Seq studies have mapped against a set of “all possible” splice junctions, consisting of all possible combinations of exons from a given annotation (as an example, see [70]), whereas in contrast we have only mapped against known splice junctions. There are several reasons for our choice of mapping. First, we believe that the mouse genome and UCSC Genes are fairly well annotated at this point, such that splices occurring in the ”all possible” set and not the known set would be rare. Therefore mapping to “all possible” junctions could create false positive mappings that could be difficult to distinguish from true novel splices. Second, the set of “all possible” splice junctions is itself incomplete, since it does not include possible splices in areas of novel transcription. As this study was focused on discovering novel transcripts, and not novel isoforms of existing transcripts, we did not feel there was much information to be gained through mapping to the “all possible” set. Finally, and perhaps most importantly, our read coverage was not sufficiently high to enable transcript assembly from spliced reads alone (see ”Novel transcript assembly” section in Text S1 and Fig. S10). Thus, while we expect our mapping rates could show modest increases with a pipeline using a set of “all possible” exon junctions, we do not expect this would have a significant impact on the interpretation of our data.

Coverage of known nuclear and cytoplasmic RNAs

Perusal of the RNA-Seq data indicates there is good separation of the nuclear and cytoplasmic RNAs in each cell type. As shown in Figure S2, RNAs for protein-coding genes show expression in both cytoplasmic and nuclear libraries; this is expected, as translation takes place in the cytoplasm while transcription takes place in the nucleus. Also as expected, intron retention is higher in nuclear libraries, corresponding to transcripts that have been incompletely processed. Nuclear RNAs show substantial enrichment in the nuclear over the cytoplasmic libraries, as illustrated with AIR, a ncRNA known to be nuclear-retained and to evade splicing [71].

Coverage of housekeeping genes

To assess the depth of coverage of our RNA-Seq data, we examined coverage of known housekeeping genes, which Warrington et al. defined for human tissues and categorized into 5 expression levels (low, low-medium, medium, medium-high, and high; [72]). In this analysis we excluded all ribosomal RNAs, as reads mapping to these RNAs were filtered out in our mapping pipeline. To find mouse homologs

of these human housekeeping genes we took the representative human mRNA sequence alignments from the xenoMrna track of the mm9 assembly of the UCSC Genome Browser; the longest isoforms of the corresponding mouse UCSC Genes in these regions were taken as the mouse homologs. To measure gene expression, we calculated the RPKM (reads per kilobase of exon model per million mapped reads, [73]) for each mouse housekeeping gene for each of our pooled RNA-Seq libraries. The distributions of RPKM values for each Warrington-defined expression level category are shown in Figure S3.

As illustrated in Figure S3, we detect expression of all Warrington-defined housekeeping genes except two predicted in the medium expression level; in all library types RPKM values were 0 for each of these two genes, which may reflect a species-specific difference. Interestingly, there is a general concordance with the expression level determined by Warrington and the RPKM values observed in our RNA-Seq data. Thus our RNA-Seq data is of sufficiently high coverage to detect even lowly-expressed genes.

Comparison with neural differentiation microarray data

We compared gene expression in our RNA-seq data with that determined with microarrays. Abranches et al. [74] performed microarray analysis on neural differentiation of 46C mESCs, using a protocol modified from Ying et al. [65, 66]. Abranches' protocol differs from Ying's, and thus ours, by the use of a commercially available N2B27-based alternative media, RHB-A (listed in Abranches' paper as available from StemCellSciences Inc., UK), instead of Ying's N2B27. They report in this media cells commit to the the neural lineage faster and in greater percentage than in N2B27. Additionally Abranches et al. measure gene expression at more finely spaced timepoints in neural differentation; they define transient NPs (tNPs), neurogenic NP (nNPs), and rosettes as three different NP populations arising in their differentiation process with distinct gene expression signatures. In the Ying protocol, neural rosettes are not expected to form until after replating onto to laminin-coated dishes [65]; neural differentiation in our study was not taken this far. A comparison of the two protocols is shown in Figure S4.

Abranches et al. defined sets of genes upregulated in each of their 4 cell populations. We chose to compare our RNA-Seq results with these gene sets (ES, tNPs, nNPs, and Rosettes) to verify the quality of our neural differentiation as well as assess the completeness of transcriptome coverage in our RNA-seq data. To evaluate tissue specificity in our RNA-Seq data, we used a measure we have termed NP Specificity, based on the tissue specificity (T_s) score described previously [75, 76]. Winter et al. [75] define T_s for each gene and each tissue in an expression compendium as the gene's fractional expression in

that tissue relative to the sum of its expression in all tissues. We applied this measure to our RNA-Seq data using the RPKMs computed for each gene in each cell and compartment type (Equation S1), and examined the distribution of scores for each of the 4 gene sets described by Abranches (Fig. S5).

$$\text{NP Specificity} = \frac{\text{RPKM}_{\text{NPNuc}} + \text{RPKM}_{\text{NPCyt}}}{\text{RPKM}_{\text{NPNuc}} + \text{RPKM}_{\text{NPCyt}} + \text{RPKM}_{\text{UnNuc}} + \text{RPKM}_{\text{UnCyt}}} \tag{S1}$$

As expected, the NP specificities for the ES gene set defined by Abranches et al. are low, while the three neural precursor gene sets show much higher NP specificity (Fig. S5). Though our neural differentiation protocol and timepoints are not directly analogous, this analysis indicates genes expected to be upregulated in undifferentiated 46Cs or 46C-derived neural precursors are indeed showing their expected expression patterns in our data; further it indicates that our RNA-Seq coverage is of good enough depth to recapitulate results seen in a similar study.

Novel transcript assembly

We initially used Cufflinks (v0.8.1) to assemble transcripts from our data [77]. Cufflinks takes in read alignments and, based on overlapping reads, outputs putative full transcript models with RPKM information (note Cufflinks technically reports a revised measure termed FPKM, for fragments per kilobase of exon per million mapped fragments; this measure is applicable to both single-end and mate-paired or paired-end reads and is equivalent to RPKM for single-end reads, used in this study). While Cufflinks does an excellent job assembling transcripts with high coverage and provides a good basis for transcript assembly, for less-highly expressed genes there are as a rule some interruptions in read coverage along the transcript, leading Cufflinks to predict several short transcripts within what should be a larger single transcript. Certainly increased sequencing depth or the use of paired-end sequencing could help reduce this problem, but given the tremendous dynamic range of expression levels within a cell, one would expect gaps in coverage within transcripts may always be present to some extent in RNA-Seq experiments. Therefore we desired a meta-assembly of Cufflinks-determined transcripts, which we will hereafter refer to as “contigs”.

To address this issue, we sought to determine if there is a measurable difference in expression levels along a gene versus in two neighboring genes. We define “adjacent internal exons” as two neighboring exons within a gene, and “adjacent outer exons” as the last exon and first exon of two neighboring genes

on the same strand. Using CufflinksG, which maps reads to a gene model, we determined the RPKMs for all isoforms of all genes in UCSC Known Genes [69]. We then selected only a single isoform for each gene, choosing the isoform with the highest RPKM. We reran CufflinksG on all exons in this single-isoform gene set, and for each exon converted its RPKM value to a "RPKM percentile" value; this value indicates for each exon its percentile rank among all exons in the gene set based on its RPKM. We then plotted the distributions of the RPKM percentile differences (absolute value) between all adjacent internal exons and all adjacent outer exons, using a Cufflinks-reported RPKM of 12 or higher to consider only those exons in expressed genes and pairs of genes. We performed this analysis for each of our 4 pooled RNA-Seq libraries to find adjacent internal exons show more similarity in RPKM percentile than do adjacent outer exons (Fig. S6).

Reasoning that the Cufflinks-determined contigs represented a situation analogous to the exon analysis just described, we thought to merge neighboring Cufflinks contigs with RPKM values reminiscent of the adjacent internal, and not adjacent outer, exons in the above analysis. The analysis in Figure S6 treats all adjacent internal exon pairs independently, regardless of gene of origin, but when choosing a cutoff to link contigs into full transcripts (analogous to linking exons into complete genes), we wanted to consider the average RPKM percentile difference across the adjacent internal pairs within a gene. This distribution is shown in Figure S7, and Figure S8 shows in more detail the distributions of the RPKM percentile differences for adjacent outer exons. Together Figures S7 and S8 suggest cutoffs can be chosen to include the values seen for most adjacent internal exon pairs while excluding the values observed for most adjacent outer exon pairs.

Additionally, we considered the length of sequence over which to allow neighboring contig joining. We examined the distribution of intron lengths, considering all unique introns in UCSC Known Genes, and compared this to the distribution of "gene spacers," which we define as the intergenic regions between two neighboring transcripts on the same strand. We found choosing a length of 11 kb would incorporate 90% of known intron lengths, while including only 23% of known gene spacers. Based off the analyses in Figures S7-S8, we first merged any Cufflinks transcripts with an RPKM percentile difference of 10 or less that were within 11 kb of each other and on the same strand. However, after inspection of the resulting novel merged transcripts, we modified this to merge Cufflinks transcripts within an RPKM percentile difference of 5 or less, keeping the same 11 kb maximum distance requirement. Using this method we sharply reduced the number of transcript fragments per UCSC Known Gene while reducing the number

of bad joins, shown in Figure S9 and Table S2. In Figure S10, we show an example of Cufflinks output at Nestin, an undifferentiated ES cell marker, in undifferentiated nuclear RNA, along with merged Cufflinks output using each of the above criteria.

We determined the analogous transcripts across the RNA-Seq libraries simply by overlapping the merged Cufflinks contains in a given library type with all other library types. To determine novel transcripts, we compared our merged Cufflinks contigs against UCSC Known Genes. This analysis returned a set of 5127 novel intergenic transcripts greater than 200 bases long from the merged Cufflinks output allowing an RPKM percentile difference of 10; in the merged Cufflinks set allowing an RPKM percentile difference of 5, 6809 such novel transcripts were returned. The 18 novel enhancer-associated transcripts described in the main text are indicated in Table S3.

Overexpression of Zmynd8as does not affect enhancer activity

We tested if the addition of Zmynd8as cDNA could increase the enhancer activity of its P300 site in undifferentiated mESCs or day 5 NPs via luciferase assay. Regions spanning the Zmynd8as P300 site were cloned as described in Methods in the main text. For these overexpression luciferase experiments, we introduced our enhancer regions upstream of luciferase in our TK-pGL4.12 vector; to do this we inserted a Gateway rfA cassette (Invitrogen) directly upstream of the TK promoter at the HindIII site using the Gateway Conversion System (Invitrogen) and transferred our enhancer clones from pENTR-D/TOPO to this vector using the Gateway LR system (Invitrogen). We additionally cloned Zmynd8as cDNA and its reverse complement into an expression vector under the control of a CMV promoter (see Methods in main text). Cells were transfected with an 8:1:1 mass ratio of enhancer construct:cDNA construct:pRL-TK (Promega) using Lipofectamine 2000 (Invitrogen) according to the manufacturer's instructions. pEGFP-N1 (Clontech) was also used as a control. Cells were assayed approximately 24 hours later with the Dual-Luciferase System (Promega), and luciferase activity was measured in a Victor Light Luminescence Counter (Perkin-Elmer); three technical replicates were measured for each biological replicate. In figures, luciferase activity (ratio of firefly counts per second/renilla counts per second) for each enhancer construct is shown relative to the activity observed for TK-pGL4.12 (ratio of enhancer construct luciferase activity: TK-pGL4.12 activity). Error bars represent 95% confidence intervals over the three biological replicates. All experiments were repeated at least twice.

As illustrated in Figure S13, the strong enhancer activity of the Zmynd8as P300 site observed in

undifferentiated mESCs is largely ablated in day 5 NPs, consistent with the broad loss of H3K4Me1 across this region in neural precursors in Mikkelsen's study ([78]; Figs. 2, S11) and the loss of P300 enrichment determined by ChIP-qPCR. Further, the exogenous expression of Zmynd8as does not appear to have an effect on the activity of the enhancer. This fact is especially telling in undifferentiated mESCs, which by nature express any other factors that might be needed should such an interaction occur endogenously. Thus it appears Zmynd8as does not behave in the same manner as the ncRNA Evf-2 [79], and does not increase enhancer activity at the P300 site.

Zmynd8 and Brd1 functional domains and the impacts of the short isoforms

Zmynd8 has been implicated in chromatin silencing and transcriptional repression [80, 81], particularly of neuronal genes in non-neuronal lineages. The full-length Zmynd8 protein contains 4 functional domains (Fig. S14A). At the N-terminus is a zinc finger domain, which has hits to zinc finger RING-, FYVE-, and PHD-type domains in Interpro [82, 83], in the middle are a bromodomain and a PWWP domain, and at the C terminus is a MYND domain. Additionally, Zmynd8 contains a domain of unknown function. The short isoform of Zmynd8 in the UCSC Genome Browser is predicted to be a target of nonsense-mediated decay (NMD, reviewed in [84]), because its final splice site occurs downstream of the termination codon of the ORF. Notably, this final splice site encompasses a SINE element. The short Zmynd8 isoform we find differs slightly at this apparent final splice junction, with the apparent 5' splice site occurring 1 base downstream of the site in UCSC Genes, for a non-canonical UU-AG intron that surrounds the SINE exactly. Further analysis of the genomic DNA in this region reveals the 46C cell line contains an allele lacking this SINE, and thus the apparent final splice site in our observed Zmynd8-short presumably does not represent an intron, but rather a structural difference between the 46C genome and the reference mouse genome. Without this final splice site, Zmynd8-short is not expected to be subject to NMD. Moreover, the high levels of the short isoform in the cytoplasm (Figs. 2A, S11) suggest this transcript is not a target of NMD, widely known as a speedy process. The expected protein domains if the short isoform of Zmynd8 is in fact translated are shown in Figure S14A.

Brd1 is reported to act in a complex with HBO1, a MYST histone acetyltransferase, to establish acetylation of histone 3 lysine 14 (H3K14Ac) at developmental regulator genes [85]. The full-length Brd1 protein contains 5 functional domains [82, 83]: an N-terminal enhancer of polycomb-like (EPL) domain, two zinc finger domains of RING/FYVE/PHD-type, a bromodomain, and a C-terminal PWWP domain

(Fig. S14B). The truncated Brd1 protein from the short isoform lacks the PWWP domain (Fig. S14B).

References

65. Ying QL, Smith AG (2003) Defined conditions for neural commitment and differentiation. Methods Enzymol 365: 327-41.

66. Ying QL, Stavridis M, Griffiths D, Li M, Smith A (2003) Conversion of embryonic stem cells into neuroectodermal precursors in adherent monoculture. Nat Biotechnol 21: 183-6.

67. Stavridis MP, Smith AG (2003) Neural differentiation of mouse embryonic stem cells. Biochem Soc Trans 31: 45-49.

68. Underwood JG, Uzilov AV, Katzman S, Onodera CS, Mainzer JE, et al. (2010) FragSeq: transcriptome-wide RNA structure probing using high-throughput sequencing. Nat Methods 7: 995–1001.

69. Hsu F, Kent WJ, Clawson H, Kuhn RM, Diekhans M, et al. (2006) The UCSC Known Genes. Bioinformatics 22: 1036–1046.

70. Ramani AK, Calarco JA, Pan Q, Mavandadi S, Wang Y, et al. (2011) Genome-wide analysis of alternative splicing in Caenorhabditis elegans. Genome Res 21: 342–348.

71. Seidl CIM, Stricker SH, Barlow DP (2006) The imprinted Air ncRNA is an atypical RNAPII transcript that evades splicing and escapes nuclear export. EMBO J 25: 3565–3575.

72. Warrington JA, Nair A, Mahadevappa M, Tsyganskaya M (2000) Comparison of human adult and fetal expression and identification of 535 housekeeping/maintenance genes. Physiol Genomics 2: 143–147.

73. Mortazavi A, Williams B, McCue K, Schaeffer L, Wold B (2008) Mapping and quantifying mammalian transcriptomes by RNA-Seq. Nat Methods 5: 621–628.

74. Abranches E, Silva M, Pradier L, Schulz H, Hummel O, et al. (2009) Neural differentiation of embryonic stem cells in vitro: a road map to neurogenesis in the embryo. PLoS One 4: e6286.

75. Winter EE, Goodstadt L, Ponting CP (2004) Elevated rates of protein secretion, evolution, and disease among tissue-specific genes. Genome Res 14: 54–61.

76. Marques AC, Ponting CP (2009) Catalogues of mammalian long noncoding RNAs: modest conservation and incompleteness. Genome Biol 10: R124.

77. Trapnell C, Williams BA, Pertea G, Mortazavi A, Kwan G, et al. (2010) Transcript assembly and quantification by RNA-Seq reveals unannotated transcripts and isoform switching during cell differentiation. Nat Biotechnol 28: 511–515.

78. Mikkelsen TS, Ku M, Jaffe DB, Issac B, Lieberman E, et al. (2007) Genome-wide maps of chromatin state in pluripotent and lineage-committed cells. Nature 448: 553–560.

79. Feng J, Bi C, Clark BS, Mady R, Shah P, et al. (2006) The Evf-2 noncoding RNA is transcribed from the Dlx-5/6 ultraconserved region and functions as a Dlx-2 transcriptional coactivator. Genes Dev 20: 1470–1484.

80. Zeng W, Kong Q, Li C, Mao B (2010) Xenopus RCOR2 (REST corepressor 2) interacts with ZMYND8, which is involved in neural differentiation. Biochem Biophys Res Commun 394: 1024–1029.

81. Poleshko A, Einarson MB, Shalginskikh N, Zhang R, Adams PD, et al. (2010) Identification of a functional network of human epigenetic silencing factors. J Biol Chem 285: 422–433.

82. Hunter S, Apweiler R, Attwood TK, Bairoch A, Bateman A, et al. (2009) InterPro: the integrative protein signature database. Nucleic Acids Res 37: D211-5.

83. Quevillon E, Silventoinen V, Pillai S, Harte N, Mulder N, et al. (2005) InterProScan: protein domains identifier. Nucleic Acids Res 33: W116-20.

84. Nicholson P, Yepiskoposyan H, Metze S, Zamudio Orozco R, Kleinschmidt N, et al. (2010) Nonsense-mediated mRNA decay in human cells: mechanistic insights, functions beyond quality control and the double-life of NMD factors. Cell Mol Life Sci 67: 677–700.

85. Mishima Y, Miyagi S, Saraya A, Negishi M, Endoh M, et al. (2011) The Hbo1-Brd1/Brpf2 complex is responsible for global acetylation of H3K14 and required for fetal liver erythropoiesis. Blood 118: 2443–2453.
