# Supplementary figures and images for: Gene Isoform Specificity through Enhancer-Associated Antisense Transcription
Source: PLoS One. 2012 Aug 24;7(8):e43511. doi: 10.1371/journal.pone.0043511 (PMC3427357; doi:10.1371/journal.pone.0043511)

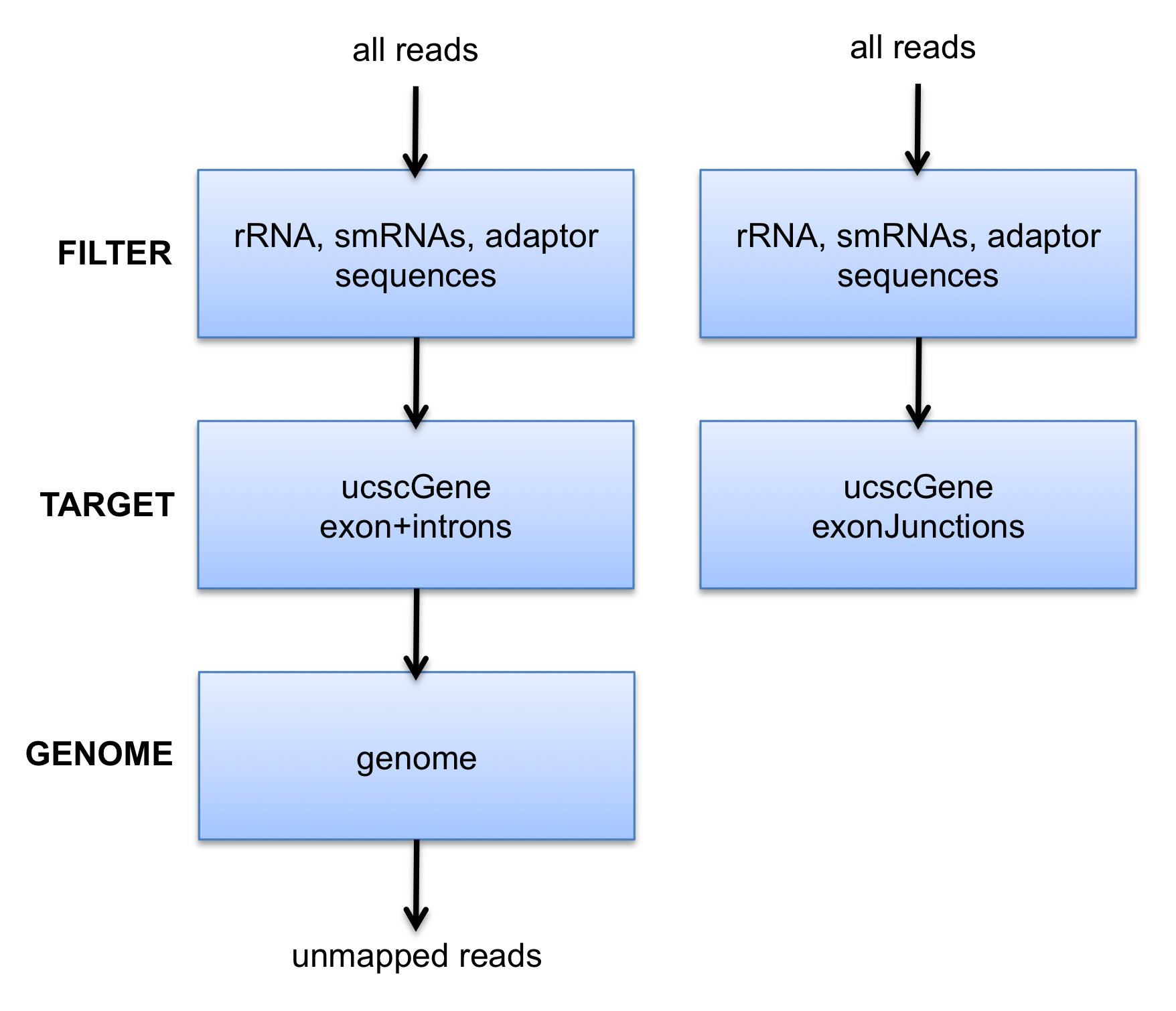

Supplement: Figure S1 — Schematic for intial mapping of RNA-Seq libraries. Mapping was performed in stages with the SOLiD Corona pipeline; see Text S1. (TIF) [file pone.0043511.s002.tif]

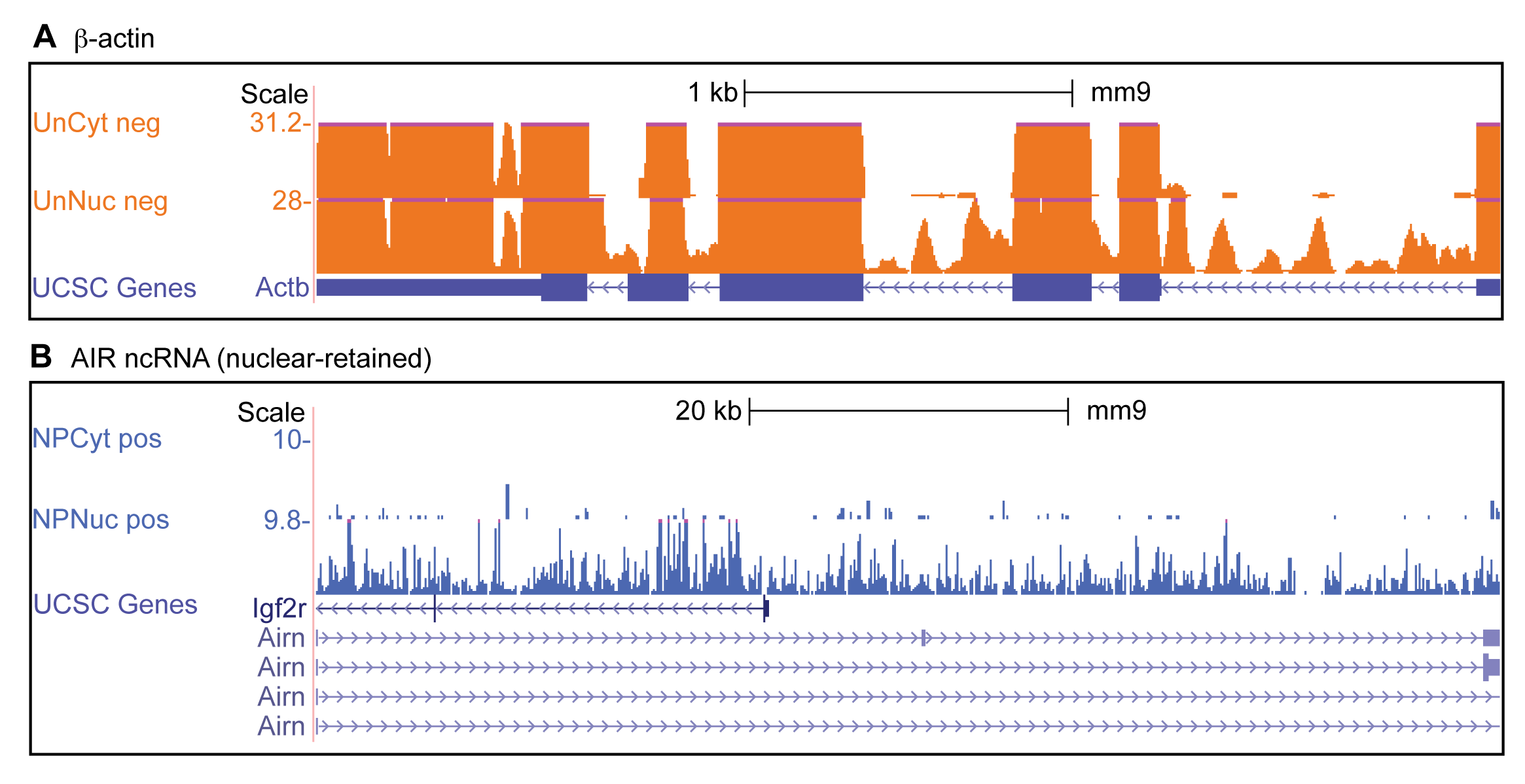

Supplement: Figure S2 — Coverage of known cytoplasmic and nuclear RNAs in RNA-Seq libraries. (A) RNA-Seq coverage tracks for -Actin, a known cytoplasmic RNA. In both libraries, coverage in exons greatly exceeds coverage in introns, but the nuclear library has greater intronic coverage than the cytoplasmic library. (B) Coverage tracks for AIR, a ncRNA known to be nuclear-retained and to evade splicing [61]. Coverage track heights (in number of reads) are indicated to the immediate left of each coverage track and are scaled according to the number of reads mapped for each RNA-Seq library. Pink coloring at the top of a coverage track indicates the number of reads mapping at that particular location exceeds the range of the track. Note track heights for panel (A) were chosen to highlight the intronic coverage observed in the nuclear library. See Text S1. (TIF) [file pone.0043511.s003.tif]

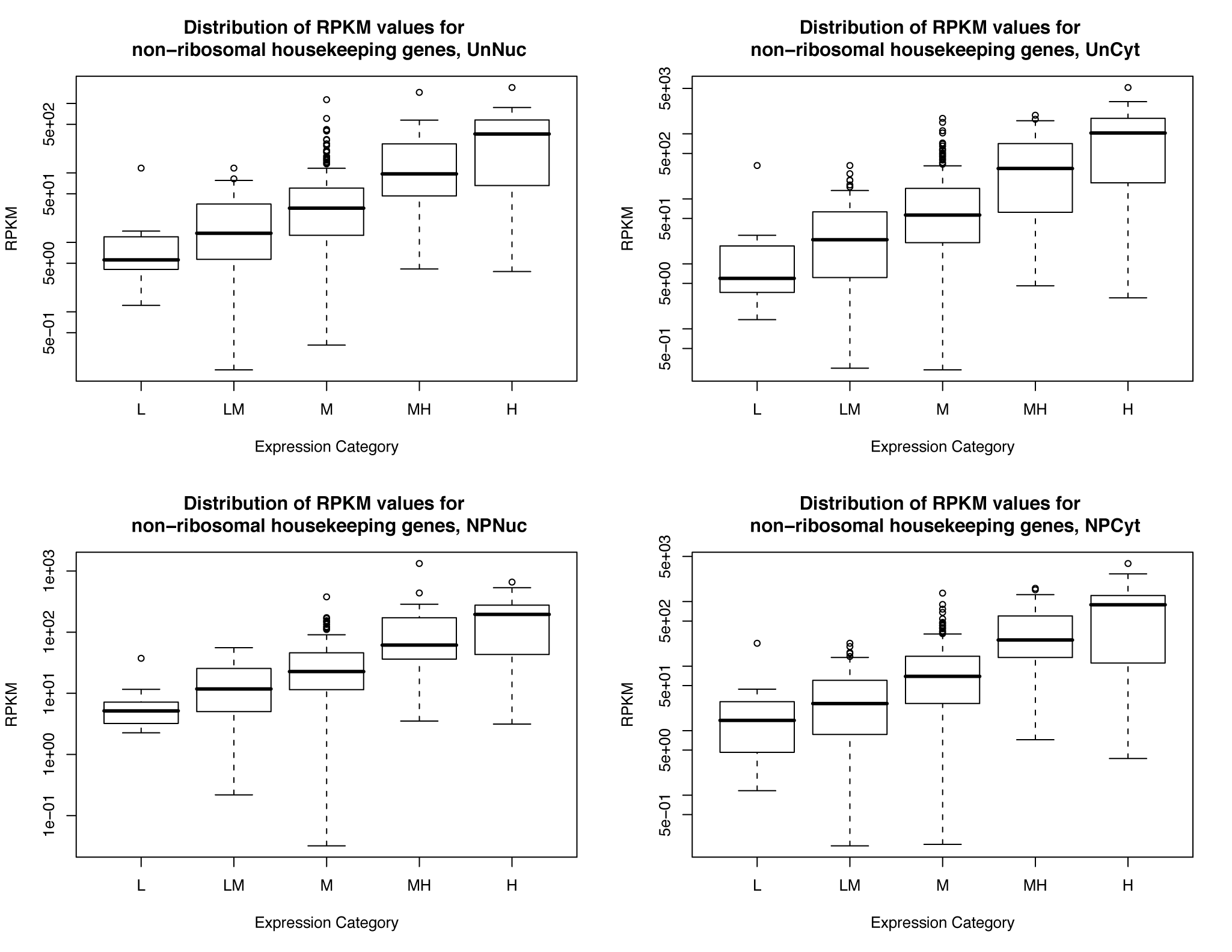

Supplement: Figure S3 — Coverage of known housekeeping genes in RNA-Seq libraries. Distributions of RPKM values for each expression level category defined by Warrington et al. [16] are shown for each RNA-Seq library type. L, low, consisting of 11 genes; LM, low-medium, 89 genes; M, medium, 230 genes (2 genes with RPKM values of 0 in all library types omitted); MH, medium-high, 22 genes; H, high, 15 genes. Data is presented in modified boxplot format. Lower and upper boundaries of boxes represent data values at the first and third quartiles, respectively. Inner bold lines indicate median data points. Whiskers extend no more than 1.5 times the interquartile distance from the first and third quartiles and represent the lowest and highest data points within this range, respectively. All other data points are plotted as outliers with open circles. See Text S1. (TIF) [file pone.0043511.s004.tif]

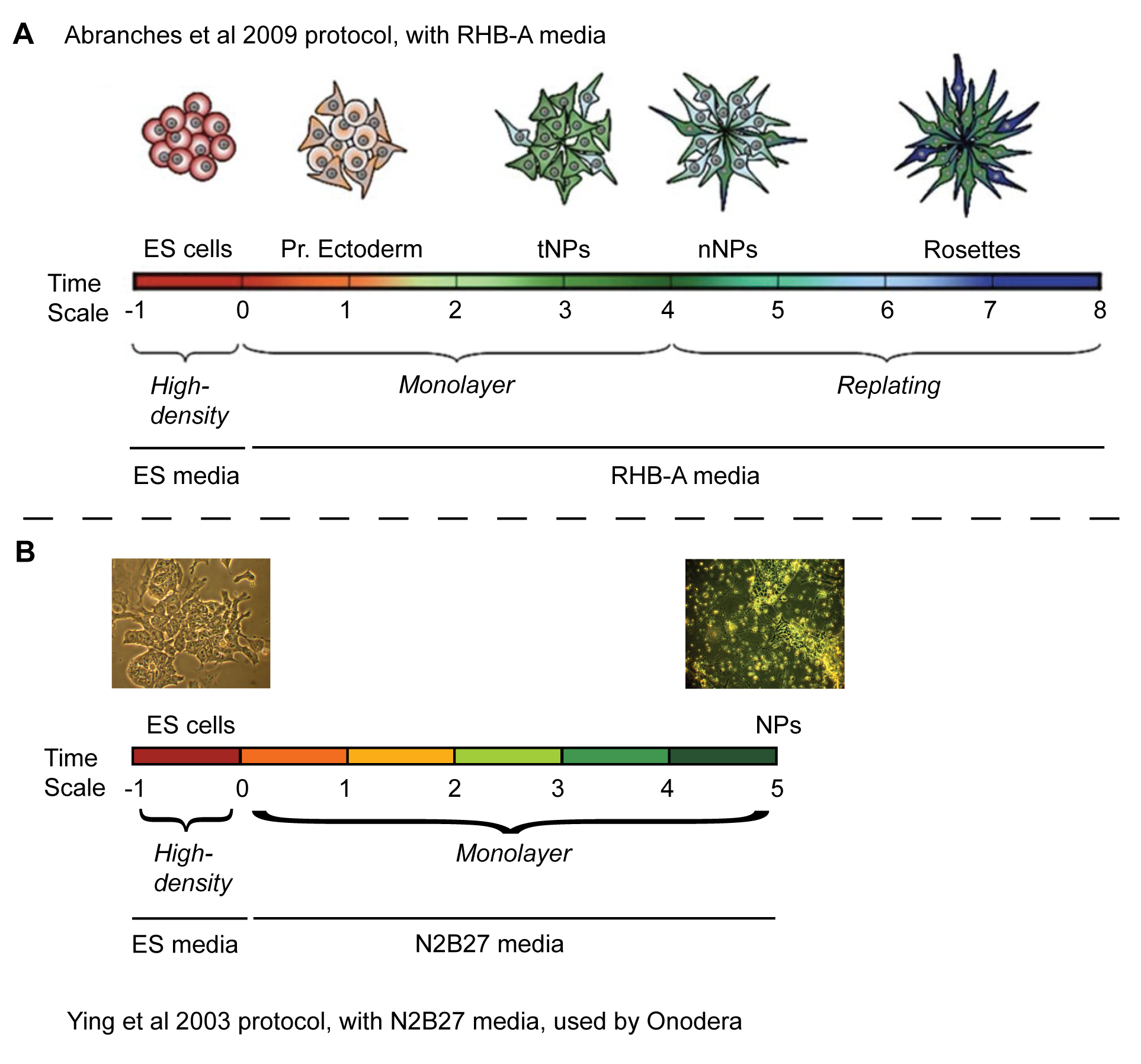

Supplement: Figure S4 — Comparison of neural differentiation protocols. Comparision of neural differentiation protocols used in Abranches et al. 2009 [17] (A), and this study (B). Image modified from [17]. See Text S1. (TIF) [file pone.0043511.s005.tif]

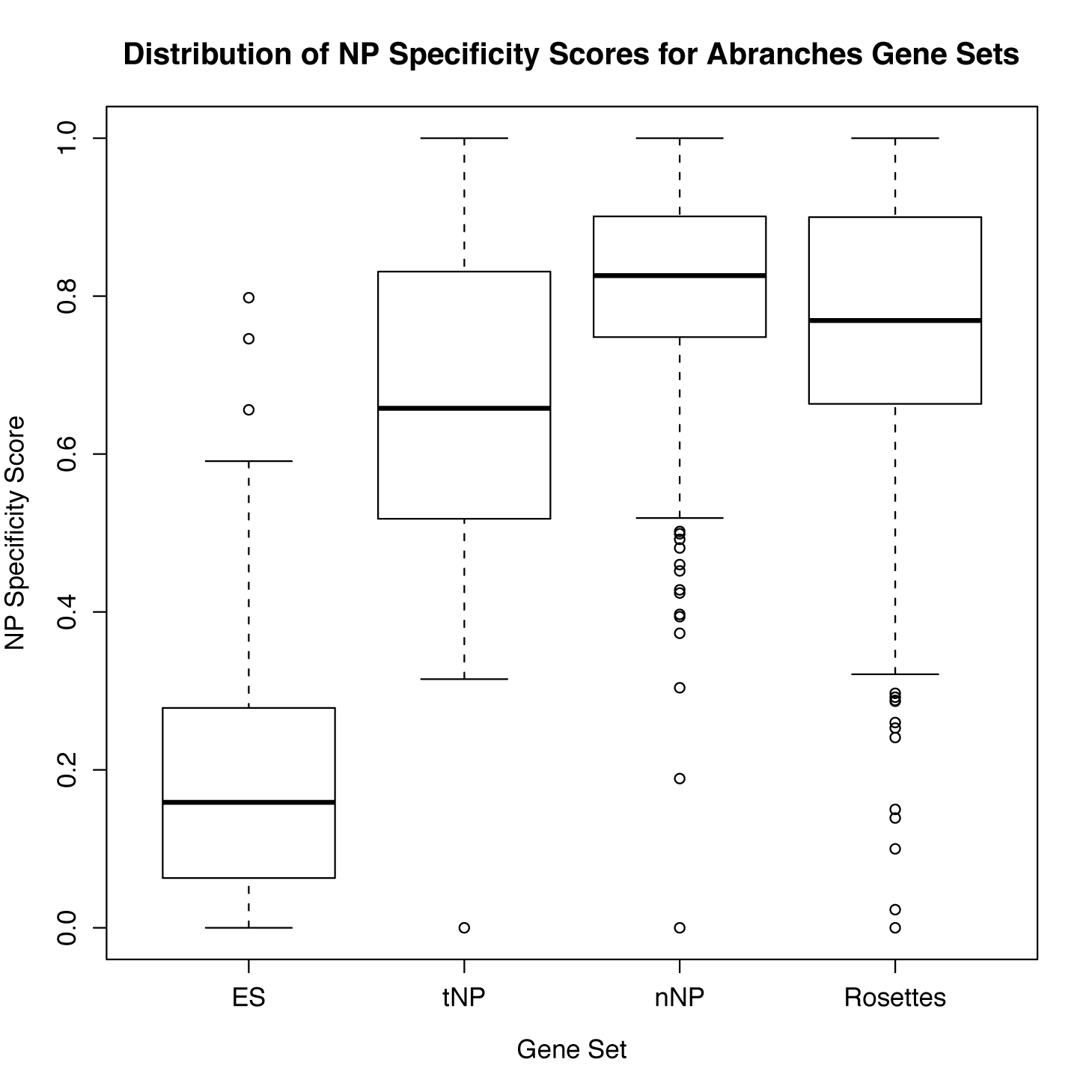

Supplement: Figure S5 — NP specificity distributions for Abranches et al. gene sets. As a measure of tissue specificity, the NP specificity scores for the genes reported upregulated in each cell type by Abranches et al. [17] are shown. See Text S1 for discussion of NP specificity. Modified boxplots are shown as in Figure S3. (TIF) [file pone.0043511.s006.tif]

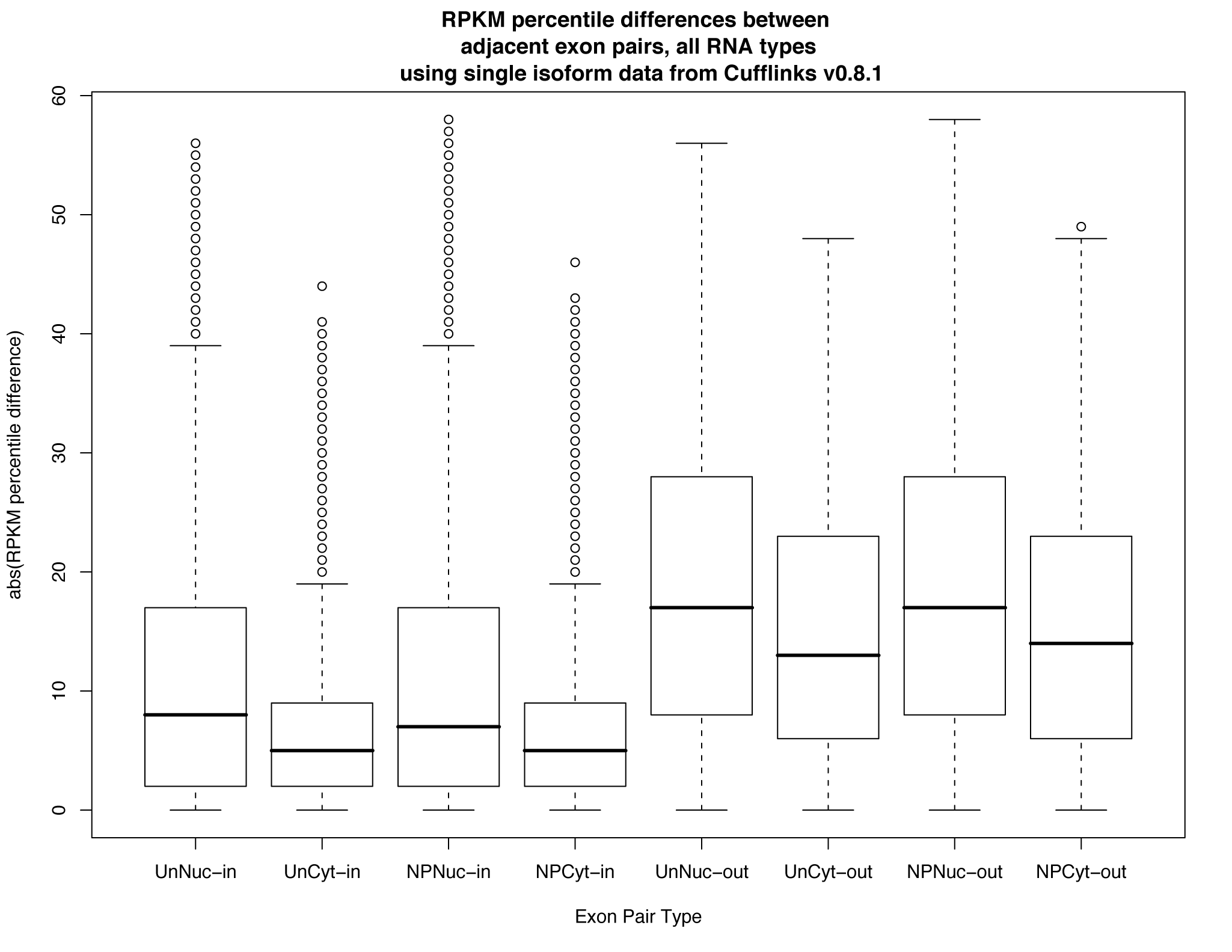

Supplement: Figure S6 — Differences in expression values for adjacent internal vs. adjacent outer exons. Expression value differences are shown as the absolute value of the RPKM percentile differences between the two exons. “in,” internal; “out,” outer. Modified boxplots are shown as in Figure S3. See Text S1. (TIF) [file pone.0043511.s007.tif]

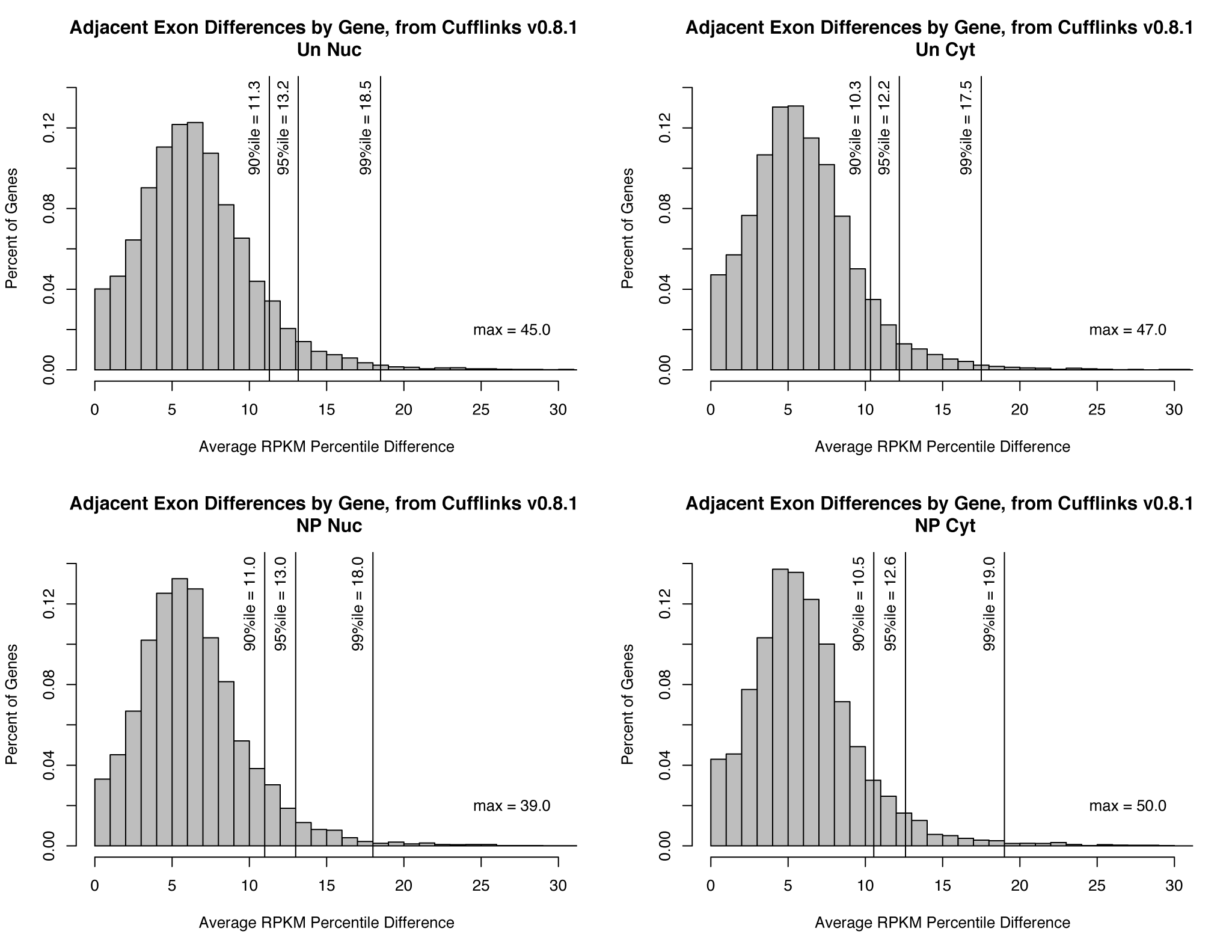

Supplement: Figure S7 — Distribution of RPKM percentile differences (averages per gene) in adjacent internal exons. The 90th, 95th, and 99th percentile values are indicated for each RNA type. See Text S1. (TIF) [file pone.0043511.s008.tif]

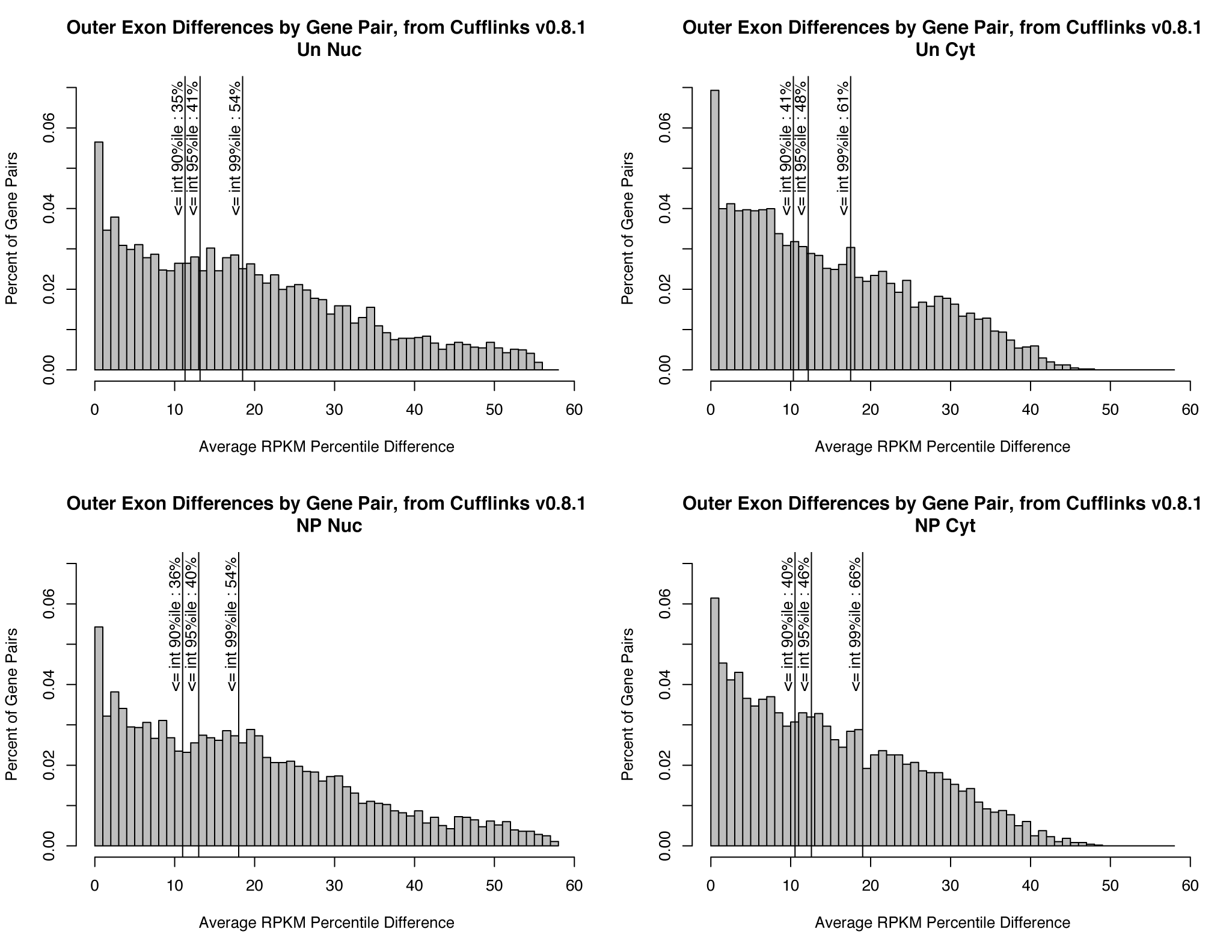

Supplement: Figure S8 — Distribution of RPKM percentile differences in adjacent outer exons. The 90th, 95th, and 99th percentile values for the adjacent internal exons shown in Figure S7 are plotted over top; the percentage of adjacent outer exons with values less than or equal to these values are indicated. See Text S1. (TIF) [file pone.0043511.s009.tif]

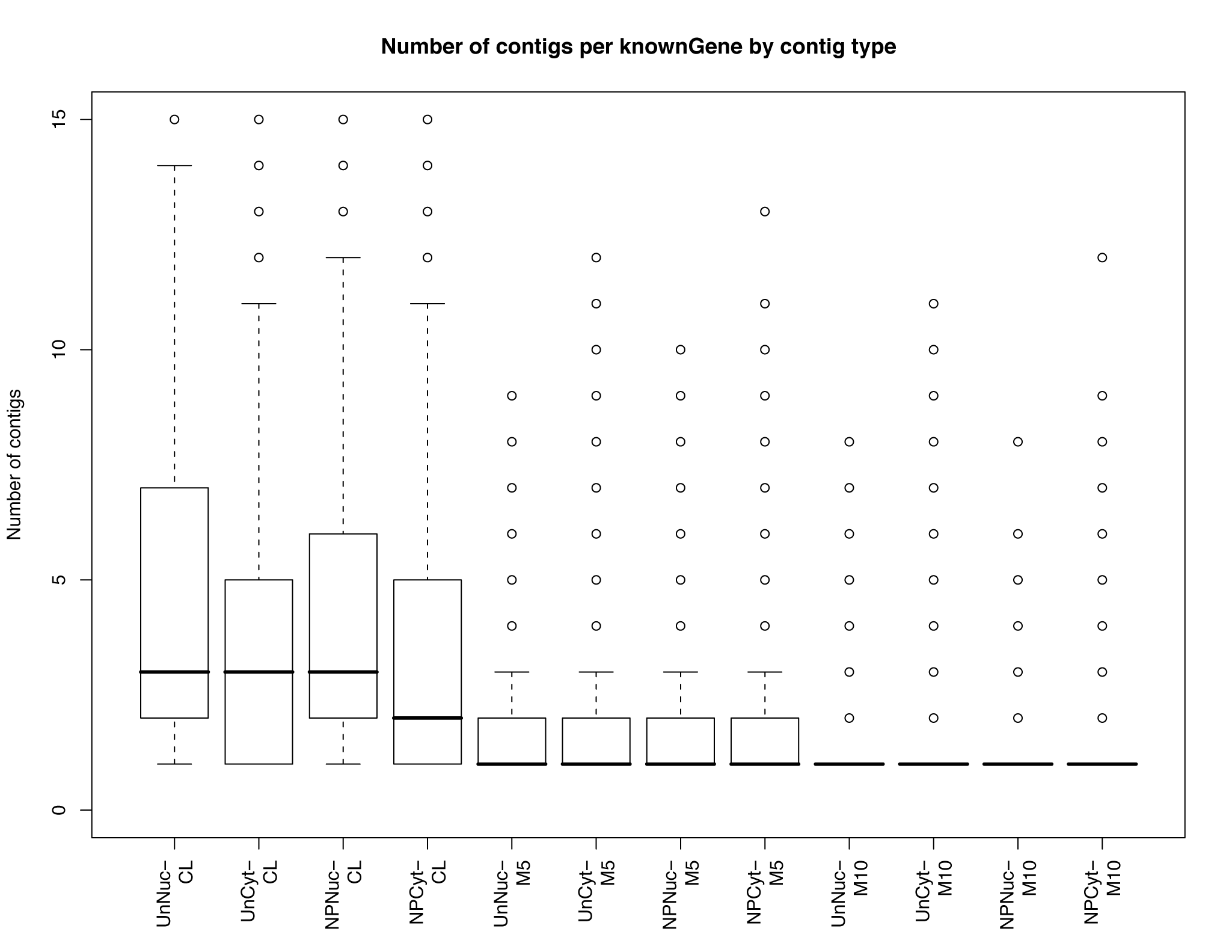

Supplement: Figure S9 — Number of fragments per UCSC Known Gene before and after merging Cufflinks transcripts. “CL,” Cufflinks transcripts with no merging; “M5,” merged Cufflinks transcripts allowing an RPKM percentile as great as 5; “M10,” merged Cufflinks transcripts allowing an RPKM percentile difference as great as 10. In merged cases, an “intron length” of up to 11 kb was allowed. Modified boxplots are shown as in Figure S3. See Text S1. (TIF) [file pone.0043511.s010.tif]

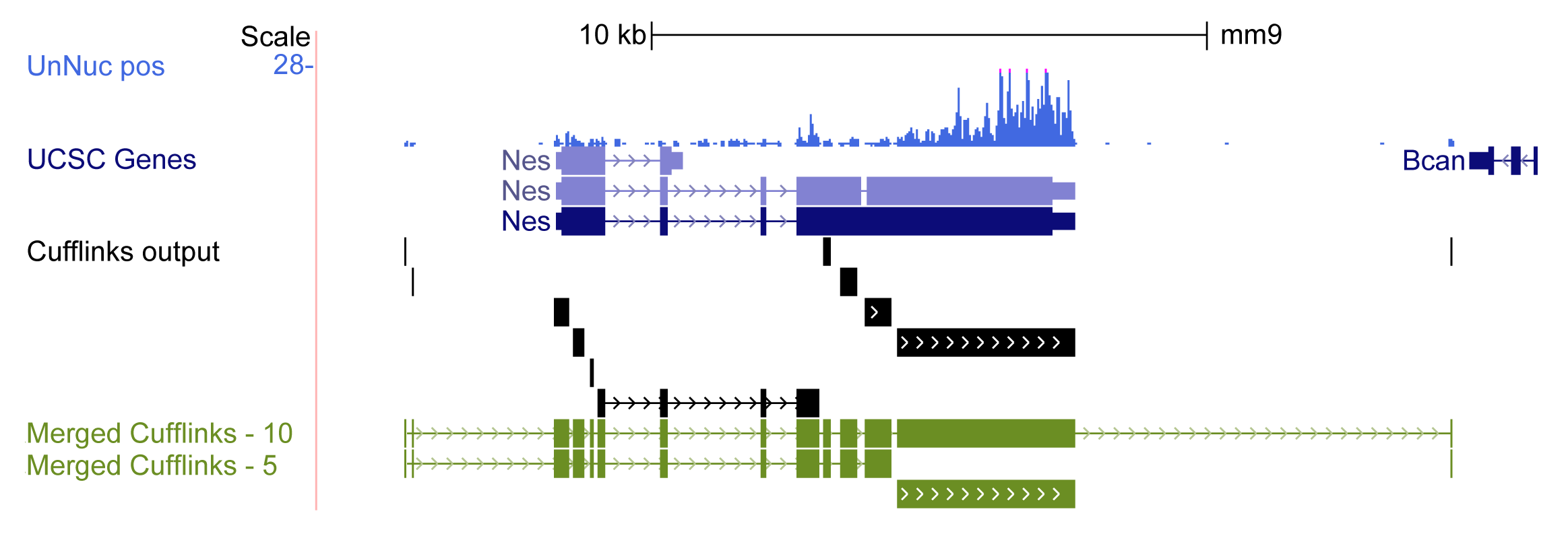

Supplement: Figure S10 — Merged Cufflinks transcripts at Nestin in undifferentiated nuclear library. RNA-Seq read coverage for Nestin is shown in the first track, above UCSC Genes, second track. Cufflinks (v0.8.1) output is third track from top; note Cufflinks predicts several fragmented transcripts along Nestin. Fourth track from top, merged Cufflinks output allowing an RPKM percentile difference of 10 and an “intron length” of 11 kb; sixth track from top, merged Cufflinks output allowing an RPKM percentile difference of 5 and an “intron length” of 11 kb. See Text S1. (TIF) [file pone.0043511.s011.tif]

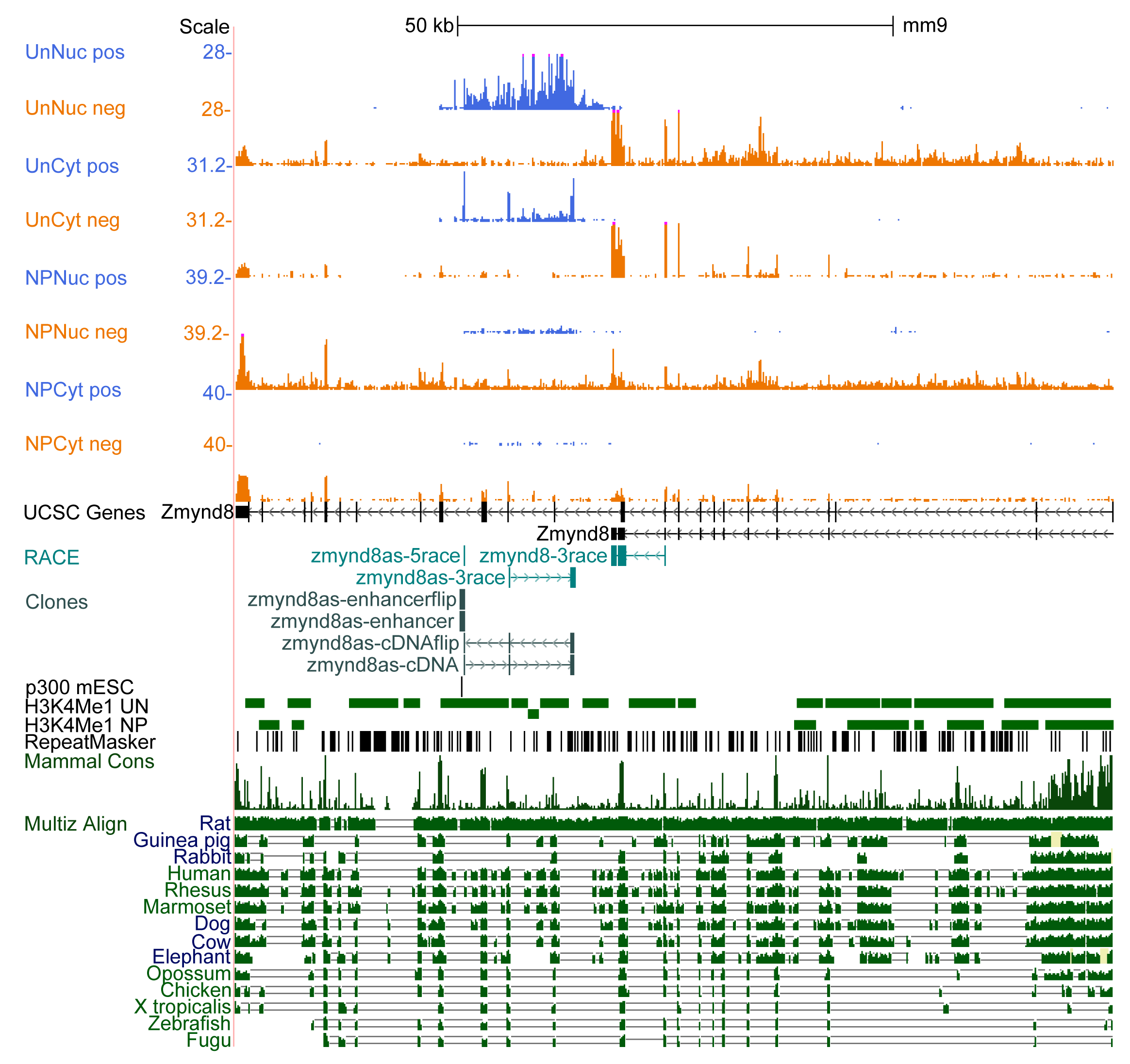

Supplement: Figure S11 — Zmynd8as and Zmynd8-short transcript structures. Structures determined with 5′ and 3′ RACE [26], [27] on undifferentiated mESC nuclear and cytoplasmic RNA; RACE products are indicated in the teal “RACE” track. Note that the apparent final splice site in the zmynd8-3race product does not represent a true intron, but a structural difference (gap) in the 46C genome relative to the reference mouse genome; see Text S1. Immediately below are shown cDNA and enhancer clones generated for Zmynd8as experiments (“Clones” track). Repeat elements are shown in the RepeatMasker track [62], second from bottom. All other tracks are as in Figure 2 of the main text: blue coverage tracks indicate the number of uniquely placed unspliced reads mapped per base on the positive strand; orange coverage tracks indicate the same for the negative strand. Coverage track heights (in number of reads) are indicated to the immediate left of each coverage track and are scaled according to the number of reads mapped for each RNA-Seq library. Also shown are UCSC Known Genes [28], along with P300 binding data (ChIP-Seq peaks) from undifferentiated mESCs [19], H3K4Me1 data (ChIP-Seq peaks) from mESCs and NPs [23], mammalian conservation (PhastCons) [58], [59], and alignments against several other species (Multiz) [63]. Known gene Zmynd8 is annotated on the negative strand, with both short and long isoforms reported; positive strand (antisense) transcription is seen in undifferentiated cytoplasmic and undifferentiated nuclear RNA. (TIF) [file pone.0043511.s012.tif]

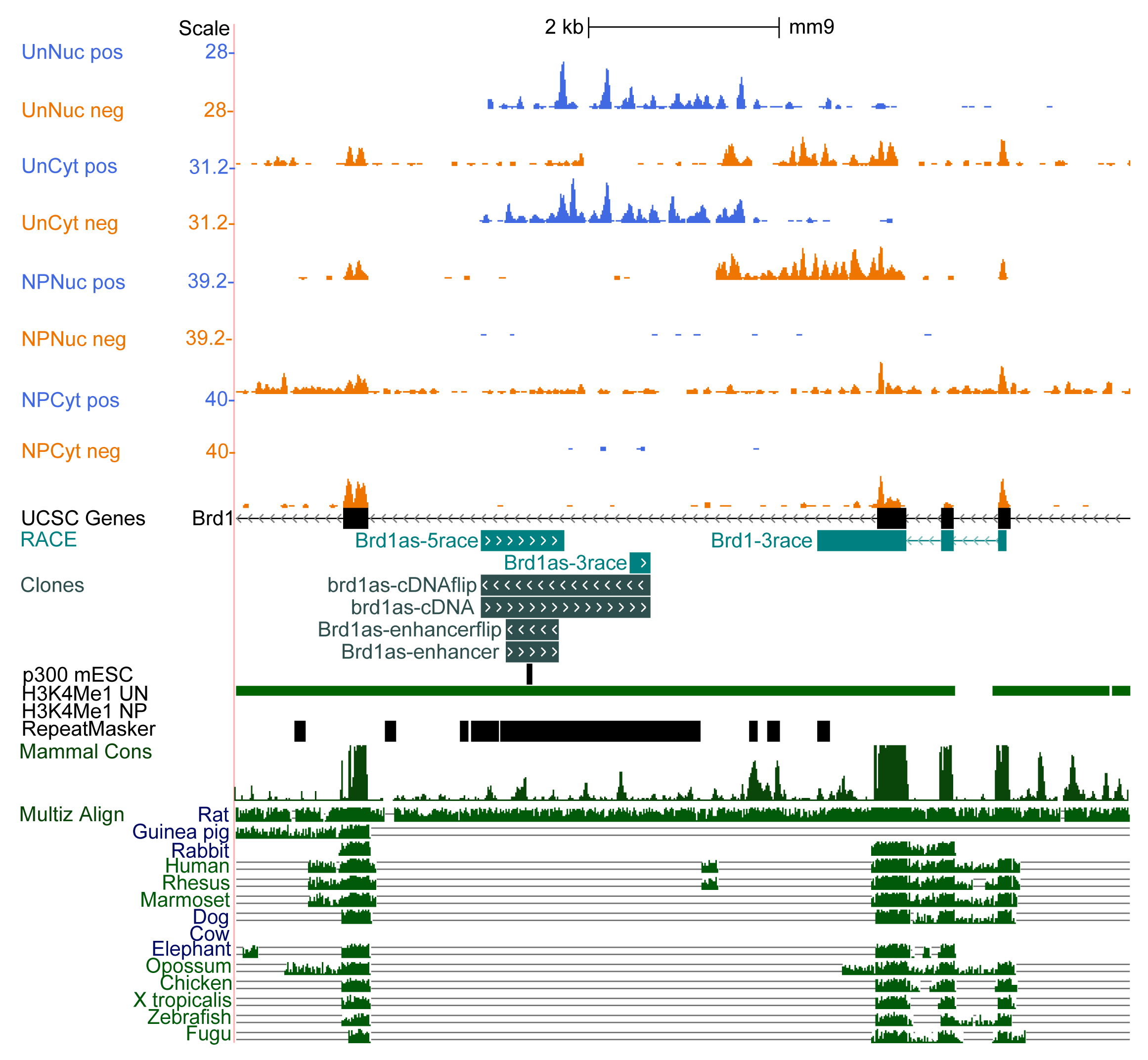

Supplement: Figure S12 — Brd1as and Brd1-short transcript structures. Structures determined with 5′ and 3′ RACE [26], [27] on undifferentiated mESC nuclear and cytoplasmic RNA; RACE products are indicated in the teal “RACE” track. Immediately below are shown cDNA and enhancer clones generated for Brd1as experiments (“Clones” track). Repeat elements are shown in the RepeatMasker track [62] second from bottom; interestingly Brd1as originates from a MaLR LTR. All other tracks are as in Figure 2 of the main text: blue coverage tracks indicate the number of uniquely placed unspliced reads mapped per base on the positive strand; orange coverage tracks indicate the same for the negative strand. Coverage track heights (in number of reads) are indicated to the immediate left of each coverage track and are scaled according to the number of reads mapped for each RNA-Seq library. Also shown are UCSC Known Genes [28], along with P300 binding data (ChIP-Seq peaks) from undifferentiated mESCs [19], H3K4Me1 data (ChIP-Seq peaks) from mESCs and NPs [23], mammalian conservation (PhastCons) [58], [59], and alignments against several other species (Multiz) [63]. Known gene Brd1 is annotated on the negative strand, with a novel 3′ end expressed in undifferentiated cells confirmed by RACE; positive strand (antisense) transcription is seen in undifferentiated cytoplasmic and undifferentiated nuclear RNA. (TIF) [file pone.0043511.s013.tif]

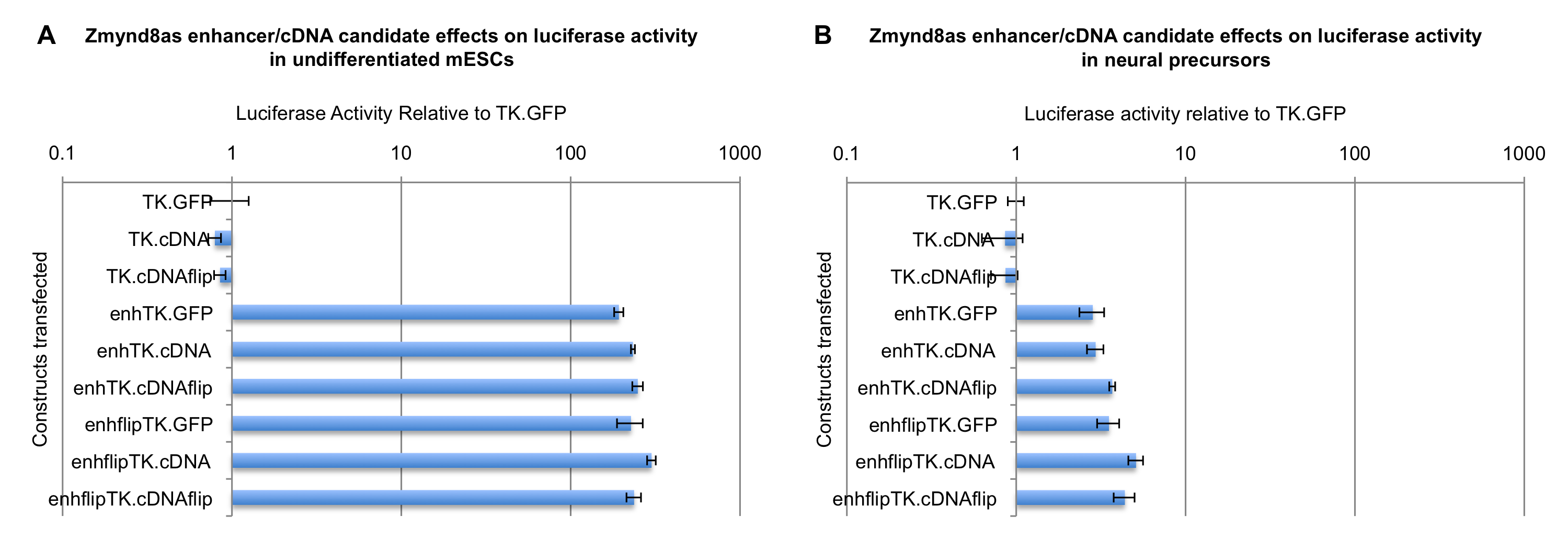

Supplement: Figure S13 — Test of enhancer activity in mESCs and NP cells with Zmynd8as cDNA. Enhancer constructs, with the Zmynd8as P300 site cloned upstream of the TK promoter, were transfected into undifferentiated mESCs (A) or NP neural precursors (B) along with a plasmid driving either Zmynd8as, its reverse complement, or GFP under a CMV promoter. TK, promoter only luciferase reporter vector; enhTK and enhflipTK, Zmynd8as P300 site cloned upstream of the TK promoter and luciferase reporter, in forward and reverse orientations, respectively; GFP, GFP plasmid under control of a CMV promoter; cDNA and cDNAflip, plasmids containing Zmynd8as and its reverse complement under the control of a CMV promoter. (TIF) [file pone.0043511.s014.tif]

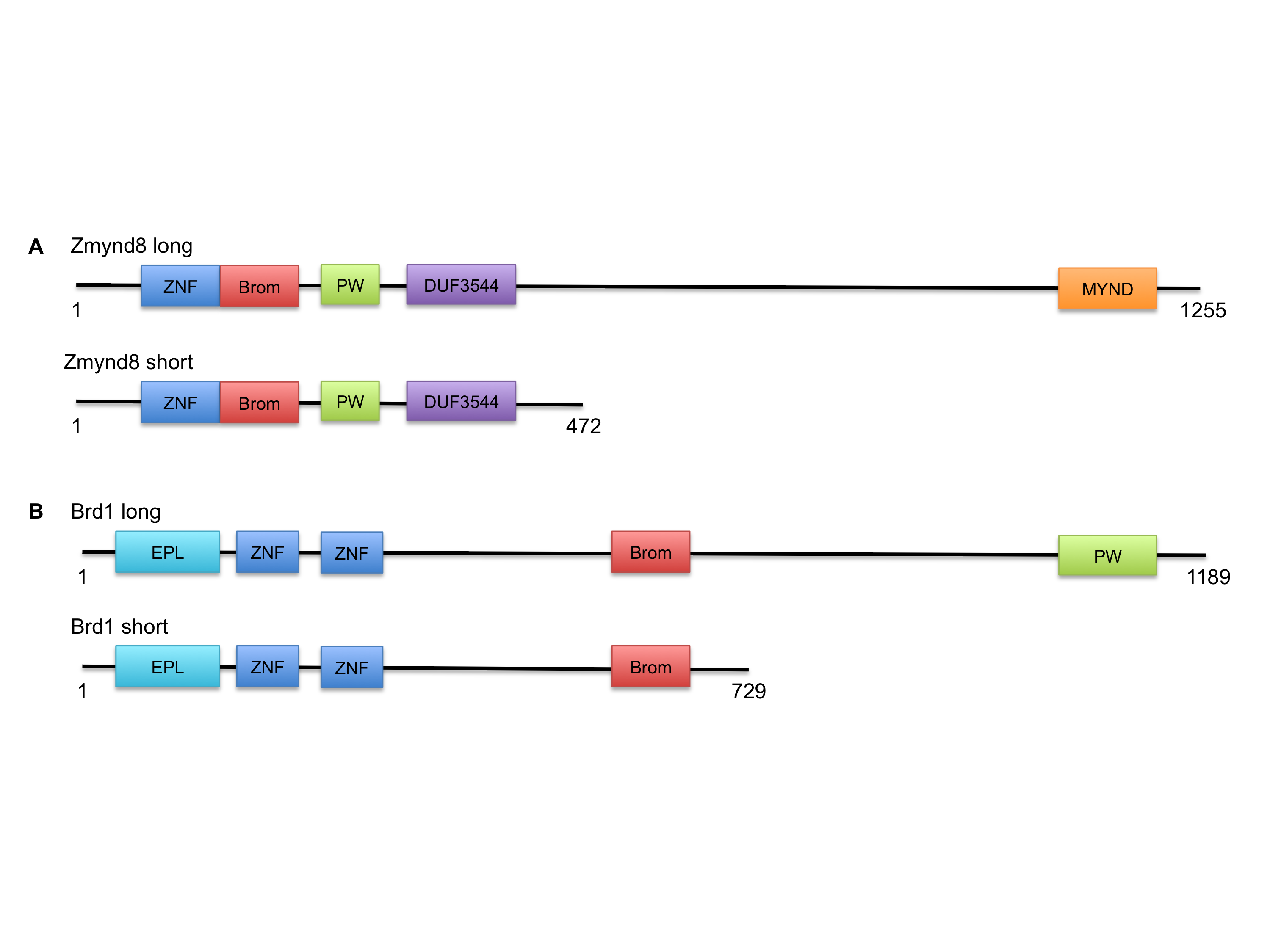

Supplement: Figure S14 — Functional domains in long and short isoforms of Zmynd8 and Brd1. Both long and short isoforms of Zmynd8 contain a zinc finger domain (ZNF; representing RING/FYVE/PHD-type domains), a bromodomain (Brom), a PWWP domain (PW), and a domain of unknown function DUF3544 (DUF); the long form also contains a zinc finger MYND-type domain (MYND). Both long and short isoforms of Brd1 contain an enhancer of polycomb-like, N-terminal domain (EPL) and two zinc finger domains and a bromodomain, as in Zmynd8; the long form also contains a PWWP domain. Numbers at bottom indicate lengths of the isoforms in amino acids. Domains determined with InterProScan [29], [30]. (TIF) [file pone.0043511.s015.tif]
