# Supplementary material for: Gene Isoform Specificity through Enhancer-Associated Antisense Transcription
Source: PLoS One. 2012 Aug 24;7(8):e43511. doi: 10.1371/journal.pone.0043511 (PMC3427357; doi:10.1371/journal.pone.0043511)
Supplement: Table S1 — RNA-Seq mapping statistics. Mapping statistics for reads from RNA-Seq experiment. Sample, RNA library information; Barcode, barcode used for sequencing; Count, total number of reads returned; Filtered, percent of reads passing filter step; Mapped, percent of reads mapped singly or multiply; Unique, percent of reads mapped uniquely. Barcodes B14–B15 were part of a separate project and were not used in this study, but are presented here for completeness of run information. “UN” indicates our undifferentiated mESC libraries, while “D5” indicates our day 5 neural precursor libraries. (PDF) [file pone.0043511.s016.pdf]

Table S1. RNA-Seq mapping statistics.

Sample	Barcode	Count	Filtered	Mapped	Unique
rna-46C-UN-CYT-PA-P1-10	B01	10892931	84.6	35.9	25.5
rna-46C-UN-CYT-PA-P1-60	B03	15035117	84.3	24.2	18.0
rna-46C-UN-CYT-PA-R3-10	B04	13066238	89.7	47.4	34.7
rna-46C-UN-NUC-PA-P1-60	B06	28325017	93.9	24.0	20.4
rna-46C-UN-NUC-PA-R3-10	B07	14738890	94.7	45.3	38.6
rna-46C-D5-CYT-PA-P1-10	B08	11224434	86.7	39.6	30.2
rna-46C-D5-CYT-PA-P1-60	B09	17000168	87.5	25.0	19.7
rna-46C-D5-CYT-PA-R3-10	B10	21650624	91.6	48.3	37.7
rna-46C-D5-NUC-PA-P1-10	B11	15663022	94.1	47.1	42.3
rna-46C-D5-NUC-PA-P1-60	B12	24108732	94.6	34.7	31.2
rna-46C-D5-NUC-PA-R3-10	B13	15223650	81.5	40.2	37.3
rna-KH2-UN-NUC-RM-R3-10	B14	12475012	84.9	36.2	32.3
rna-KH2-D5-NUC-RM-R3-10	B15	14433434	96.4	53.1	48.0
Onodera Project-Specific	B01-B13	186928823	90.2	36.5	30.1
Total	all	213837269	90.3	37.6	31.4

Mapping statistics for reads from RNA-Seq experiment. Sample, RNA library information; Barcode, barcode used for sequencing; Count, total number of reads returned; Filtered, percent of reads passing filter step; Mapped, percent of reads mapped singly or multiply; Unique, percent of reads mapped uniquely. Barcodes B14-B15 were part of a separate project and were not used in this study, but are presented here for completeness of run information. “UN” indicates our undifferentiated mESC libraries, while “D5” indicates our day 5 neural precursor libraries.
