# Supplementary material for: Gene Isoform Specificity through Enhancer-Associated Antisense Transcription
Source: PLoS One. 2012 Aug 24;7(8):e43511. doi: 10.1371/journal.pone.0043511 (PMC3427357; doi:10.1371/journal.pone.0043511)
Supplement: Table S2 — Bad joins of UCSC Known Genes in Cufflinks and merged Cufflinks output. “Bad joins” occur when two separate genes are merged into a single transcript. Cufflinks bad joins, bad joins by Cufflinks; d10, bad joins when an RPKM percentile difference of 10 is allowed for merging Cufflinks output; d5, bad joins when an RPKM percentile difference of 5 is allowed for merging Cufflinks output. (PDF) [file pone.0043511.s017.pdf]

Table S2. Bad joins of UCSC Known Genes in Cufflinks and merged Cufflinks output.

		Merged Cufflinks bad joins	
RNA Type	Cufflinks bad joins	d10	d5
UnNuc	54	3186	3365
UnCyt	55	1980	1669
NPNuc	73	3661	3828
NPCyt	83	2021	1672

"Bad joins" occur when two separate genes are merged into a single transcript. Cufflinks bad joins, bad joins by Cufflinks; d10, bad joins when an RPKM percentile difference of 10 is allowed for merging Cufflinks output; d5, bad joins when an RPKM percentile difference of 5 is allowed for merging Cufflinks output.
