# Supplementary material for: Gene Isoform Specificity through Enhancer-Associated Antisense Transcription
Source: PLoS One. 2012 Aug 24;7(8):e43511. doi: 10.1371/journal.pone.0043511 (PMC3427357; doi:10.1371/journal.pone.0043511)
Supplement: Table S3 — ncRNA candidates for study from the RNA-Seq project. Columns indicate the following information: Region, genomic coordinates for area of novel transcription on the UCSC Genome Browser mouse assembly mm9; Strand, strand of novel transcription; Nearest Gene, nearest known gene; Description, properties of novel transcription; Expression, RNA-Seq libraries containing the novel transcription; Enhancer, cell types with enhancer activity in this area, with enhancers defined from previous literature as stated in the text of this section. Two identified regions correspond to lincRNAs first reported by Guttman et al. [64]. NP, neural precursors. (PDF) [file pone.0043511.s018.pdf]

Table S3. ncRNA candidates for study from the RNA-Seq project.

Region	Strand	Nearest Gene	Description	Expression	Enhancer
chr2:20,890,433-20,892,100	+	Gm13375	transcription of enhancer, single strand	NPNuc,NPCyt	NP
chr2:84,573,971-84,585,570	-	Ypel4, mir130a	bidirectional transcription of enhancer	UnNuc,NPNuc	mESC, NP
chr2:165,618,601-165,668,700	+	Zmynd8	bidirectional transcription of enhancer	UnNuc,UnCyt	mESC
chr3:89,018,478-89,033,164	+	THBS3, mir92b	bidirectional transcription of enhancer; intronic	NPNuc	NP, forebrain
chr4:22,406,798-22,425,777	+	Oct3	transcription of enhancer, single strand	NPNuc,NPCyt	NP
chr4:99,319,831-99,326,069	-	Foxd3	bidirectional transcription at homeobox gene	UnNuc	none
chr4:123,340,299-123,362,182	+	Macf1	bidirectional transcription of enhancer; lincRNA	NPNuc	mESC, NP
chr5:123,583,162-123,592,735	-	Setd1b	transcription of enhancer, single strand; lincRNA	UnNuc,NPNuc	mESC, NP
chr6:124,806,079-124,814,476	+	Gpr162	bidirectional transcription of enhancer	NPNuc	mESC, NP
chr7:80,564,273-80,569,279	+	Rgma	transcription of enhancer, single strand	NPNuc,NPCyt	NP
chr8:91,564,336-91,582,454	-	Sall1	transcription of enhancer, single strand	UnNuc,UnCyt	mESC
chr9:39,982,808-40,052,077	-	Zfp202	transcription of enhancer, single strand	UnNuc	mESC, NP
chr10:80,869,755-80,882,086	+	Nfic	bidirectional transcription of enhancer	UnNuc,UnCyt	mESC, NP
chr13:83,878,326-83,881,072	+	C130071C03Rik	transcription of enhancer, single strand	NPNuc, NPCyt	forebrain, NP
chr15:88,538,699-88,541,837	+	Brd1	bidirectional transcription of enhancer	UnNuc,UnCyt	mESC
chr16:35,472,958-35,491,824	+	Pdia5	bidirectional transcription of enhancer	NPNuc	NP
chr17:37,103,007-37,109,588	-	RT1-M6-2 (rat)	transcription of enhancer, single strand	UnNuc,UnCyt	mESC
chr19:57,676,283-57,778,594	-	Atrnl1	bidirectional transcription of enhancer	UnNuc,NPNuc	mESC

Columns indicate the following information: Region, genomic coordinates for area of novel transcription on the UCSC Genome Browser mouse assembly mm9; Strand, strand of novel transcription; Nearest Gene, nearest known gene; Description, properties of novel transcription; Expression, RNA-Seq libraries containing the novel transcription; Enhancer, cell types with enhancer activity in this area, with enhancers defined from previous literature as stated in the text of this section. Two identified regions correspond to lincRNAs first reported by Guttman et al [64]. NP, neural precursors.
