# Supplementary material for: Gene Isoform Specificity through Enhancer-Associated Antisense Transcription
Source: PLoS One. 2012 Aug 24;7(8):e43511. doi: 10.1371/journal.pone.0043511 (PMC3427357; doi:10.1371/journal.pone.0043511)
Supplement: Table S4 — Sequences of primers, siRNAs, and ASOs used in this study. Primers were used for a variety of purposes, indicated in the “Purpose” column. For RACE primers, primer names indicate whether the primer was used in 5′ or 3′ RACE. Note forward cloning primers have “CACC” at their 5′ ends to allow directional cloning with the Invitrogen Gateway System. For ASOs, the five terminal nucleotides on each end are 2′-Omethoxyethyl nucleotides indicated as “mN,” where N is the nucleotide. The phosphothioate backbones are indicated with asterisks. (PDF) [file pone.0043511.s019.pdf]

Table S4. Sequences of primers, siRNAs, and ASOs used in this study. Primers were used for a variety of purposes, indicated in the "Purpose" column. For RACE primers, primer names indicate whether the primer was used in 5' or 3' RACE. Note forward cloning primers have "CACC" at their 5' ends to allow directional cloning with the Invitrogen Gateway System. For ASOs, the five terminal nucleotides on each end are 2'-Omethoxyethyl nucleotides indicated as "mN," where N is the nucleotide. The phosphothioate backbones are indicated with asterisks.

Primer name/target	Sequence (forward/reverse)		Purpose
Brd1as-5race-1	GCAGTCACCCAGCATAGCTGCAGCATGA		RACE
Brd1as-5race-2	CACGTGGGGATCTTGCTCAGAACTCTGA		RACE
Brd1as-3race-1	GGTCCCCCAGGTGGCACTGGCCTTGA		RACE
Brd1as-3race-2	CAGCAGCAGCGGCAGTGGAGGGATCAA		RACE
Brd1as-3race-3	GCTGCAGCTATGCTGGGTGACTGCAA		RACE
brd1-5race-1	GCTCGGGGTGGAGTTCTACTGGGTTGA		RACE
brd1-5race-2	CCCGTTTACTCCGTGAGCCGCTGGACTT		RACE
brd1-5race-3	GCGATGGGTCGCTCAGGCAGGTGCATT		RACE
brd1-5race-4	GCAGGACCGCACAGGGCTCAGCCAGTGT		RACE
Brd1-3race-0	GAGGAAACGGCTAGAAGCTCAAGGGTAT		RACE
Brd1-3race-0up	CTGGCAGCGGCTGCGACATGACCTAGA		RACE
Brd1-3race-1	GGCTGCGCGACCAGGGAGGGGTTGT		RACE
Brd1-3race-1nest	GGGTCTGACCTTGCTTCTGAGGTCATGCA		RACE
zmynd8as-5race-1	CCGAGGTGGCTAGGCGATGCTCCTAGCT		RACE
zmynd8as-5race-2	CTCGGGATCACGGGTGTACCCGGCACTT		RACE
zmynd8as-5race-3	GGCAATGGTCCTGCCAGCCTCGGGAAGA		RACE
zmynd8as-3race-1	GCTGCTGGACCGACTCGCTCAGCTCCTT		RACE
zmynd8as-3race-2	GGCTGGCAGGACCATTGCCGGAGGACAA		RACE
zmynd8as-3race-3	GGCCCAGGAGGGCAGTAAACAAGACA		RACE
zmynd8-3race-1	CTGGCCAGCGAAAGCTCTGAGGGACAA		RACE
zmynd8-3race-2	CCGGCCGCAGGATCTCCCTGTCCGACAT		RACE
Zmynd8as cDNA	CACCGTCTACATCTGCTCCTGAGCTGGGT	CCTTTATTTGTGTGTTGTGTGCTGACCTA	cloning
Zmynd8as cDNAflip	CACCCCTTTATTTGTGTGTTGTGTGCTGACCTA	GTCTACATCTGCTCCTGAGCTGGGT	cloning
Brd1as cDNA	CACCGAGTCAGTACTCTGCTAGCAGCCTTCAGAAGACGTAGA	GTTGACTGCAACAAACTTCTTTCTCCTG	cloning
Brd1as cDNAflip	CACCGTTGACTGCAACAAACTTCTTTCTCCTGG	GAGTCAGTACTCTGCTAGCAGCCTTCAGAAGACGTAGA	cloning
Zmynd8as p300 site	TGGAAATTTCTTCAGTAGGGTGA	GGCTGGTAGGTAATCTGCATTT	ChIP-qPCR
Brd1as p300 site	CTATTTTTGCCCTTGGCTGA	TTCAGCTCCATTCACTTCCA	ChIP-qPCR
Control (chr9)	GATGAAGGGGGACATCACAC	AGGATCCCATGTTTGTGAGC	ChIP-qPCR
Brd1as enhancer	CACCTGGGGAACTTGGGATTTGGA	GTACCAGCACGCCCGGAAT	cloning
Brd1as enhancer flip	CACCGTACCAGCACGCCCGGAAT	TGGGGAACTTGGGATTTGGA	cloning
Zmynd8as p300 site	CACCGCCCGGCTGTAGATCTTTTTCC	GAGGTGGCTAGGCGATGCTC	cloning
Zmynd8as p300 flip	CACCGAGGTGGCTAGGCGATGCTC	GCCCGGCTGTAGATCTTTTTCC	cloning
brd1as	TGCTGGGTGACTGCAATGGT	GGGCTTCCGCTGCAACTAGA	qRTPCR
brd1-short	GGCCTCGCCATGTTTTCACA	GCTAAGGCTGGGCCCTCAAT	qRTPCR
brd1-long	GAGTGCCTGGCCACCACAAT	TCAAATGCAATCCGCACAGC	qRTPCR
brd1-both	TGGCCATTCAGCGGAAGAAG	GCTTGCGCAGCAGCTCAATTA	qRTPCR
zmynd8as	TCAGCTCCTTCAGGTCCATC	GGCCTTTAATCCCAGCTCTC	qRTPCR
zmynd8-short	CTGGAAAAGCCAGCCCTGAA	TCATGGCTGTTCGCAGCAGT	qRTPCR
zmynd8-long	GCAGCCTGCCTATACCCCAAC	TGGCTCACAATGCACCCCTA	qRTPCR
zmynd8-both	GTTGACGCCCGTTTCTTTGG	GCTGGGATCCAGCAGCATTT	qRTPCR
gapdh	TGAGGCCGGTGCTGAGTATG	TGGTTCACACCCATCACAAACA	qRTPCR
Brd1as siRNA	CAGGUGGCACUGGCCUUGAGGCAUG		knockdown
Brd1as control (1355) siRNA	CAGCGGUCACGGUUCGAGCGGUAUG		knockdown
			Continued on next page ...

Table S4 – continued from previous page

Primer name/target	Sequence (forward/reverse)	Purpose
Zmynd8as siRNA	UGGAUAGGCACACCACGUGCAUGCG	knockdown
Zmynd8as control (554) siRNA	UGGUAAGGAUACACCACCCGUGGCG	knockdown
aso-mmBrd1as-6	mG*mG*mC*mC*mC*C*T*T*C*A*T*G*C*C*T*mC*mA*mA*mG*mG	knockdown
aso-mmZmynd8as	mG*mG*mA*mU*mA*G*G*C*A*C*A*C*C*A*C*mG*mU*mG*mC*mA	knockdown
aso-ISIS141923 (control)	mC*mC*mU*mU*mC*C*C*T*G*A*A*G*G*T*T*mC*mC*mU*mC*mC	knockdown
